# High‐Affinity Superantigen‐Based Trifunctional Immune Cell Engager Synergizes NK and T Cell Activation for Tumor Suppression

**DOI:** 10.1002/advs.202310204

**Published:** 2024-06-27

**Authors:** Yao‐An Yu, Wan‐Ju Lien, Wen‐Ching Lin, Yi‐Chung Pan, Sin‐Wei Huang, Chung‐Yuan Mou, Che‐Ming Jack Hu, Kurt Yun Mou

**Affiliations:** ^1^ Institute of Biomedical Sciences Academia Sinica Taipei 11529 Taiwan; ^2^ Doctoral Degree Program of Translational Medicine National Yang Ming Chiao Tung University and Academia Sinica Taipei 112 Taiwan; ^3^ Department of Chemistry National Taiwan University Taipei 10617 Taiwan; ^4^ Biomedical Translation Research Center Academia Sinica Taipei 11529 Taiwan

**Keywords:** cancer Immunotherapy, directed evolution, fusion protein, immune cell engager, superantigens

## Abstract

The development of immune cell engagers (ICEs) can be limited by logistical and functional restrictions associated with fusion protein designs, thus limiting immune cell recruitment to solid tumors. Herein, a high affinity superantigen‐based multivalent ICE is developed for simultaneous activation and recruitment of NK and T cells for tumor treatment. Yeast library‐based directed evolution is adopted to identify superantigen variants possessing enhanced binding affinity to immunoreceptors expressed on human T cells and NK cells. High‐affinity superantigens exhibiting improved immune‐stimulatory activities are then incorporated into a superantigen‐based tri‐functional yeast‐display‐enhanced multivalent immune cell engager (STYMIE), which is functionalized with a nanobody, a Neo‐2/15 cytokine, and an Fc domain for tumor targeting, immune stimulation, and prolonged circulation, respectively. Intravenous administration of STYMIE enhances NK and T cell recruitment into solid tumors, leading to enhanced inhibition in multiple tumor models. The study offers design principles for multifunctional ICEs.

## Graphical abstract

The Superantigen‐based Trifunctional Yeast‐display‐enhanced Multivalent Immune Cell Engager (STYMIE) integrates an engineered superantigen with enhanced immune stimulatory properties, a tumor‐targeted nanobody, an immune cell‐stimulating cytokine for prolonged immune cell survival, and an Fc domain for extended circulation. STYMIE successfully recruits NK and T cells to solid tumors, leading to substantial inhibition of tumor growth in various murine tumor models.

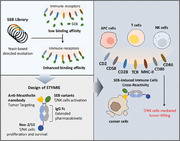

## 1 Introduction

Immune cell engagers (ICEs) present a promising therapeutic approach for redirecting immune cells for tumor suppression, and recent clinical approval of bi‐specific T cell engager (BiTE) against B cell malignancies has fueled continuing enthusiasm for multifunctional ICEs development against other oncological diseases.^[^ ^1^, ^2^, ^3^, ^4^ ^]^ Efforts to improve the treatment efficacy of ICEs against the immune‐suppressive environment of solid tumors have introduced other functional elements into fusion protein designs,^[^ ^5^, ^6^ ^]^ including Fc region for circulatory half‐life extension,^[^ ^7^, ^8^ ^]^ ligand against inhibitory molecules for T cell invigoration,^[^ ^9^, ^10^ ^]^ and cytokines for enhanced immune cell expansion.^[^ ^11^, ^12^ ^]^ However, the therapeutic efficacy of ICEs against solid tumors remains limited despite these advances. Envisioning that solid tumor treatment can benefit from multivalent immune cell recruitment^[^ ^13^ ^]^ we herein devise a multivalent ICE through directed evolution of bacterial superantigens (SAgs) for simultaneous activation of NK and T cells. Systematic screening and characterizations are performed to identify high‐affinity bacterial superantigens, which are subsequently integrated into a trifunctional ICE for intravenous anticancer immunotherapy.

SAgs are a family of secreted protein toxins primarily produced by *Staphylococcus aureus* and *Streptococcus pyogenes*. SAgs act as unconventional antigens by triggering extensive T cell expansion through the cross‐linking of T cell receptor and MHC‐II on the antigen‐presenting cells (APCs) outside the antigen‐binding groove^[^ ^14^ ^]^ It has also been reported to interact with the costimulatory receptor CD28 for T cell activation.^[^ ^15^, ^16^, ^17^ ^]^ Moreover, SAgs are known to induce cytokine secretion and activation of NK cells, γδ T cells, and invariant natural killer T cells,^[^ ^18^, ^19^ ^]^ although the specific ligand and mechanisms are unclear^[^ ^20^ ^]^ This characteristic has led to the creation of tumor‐targeted superantigens (TTSs) by linking the SAgs to tumor‐specific antibodies or ligands. In comparison to common ICEs based on CD3 antibodies, TTSs present a unique class of ICEs as they are cross‐reactive with multiple immune cell types, including T cells, antigen‐presenting cells (APCs), and NK cells.^[^ ^21^, ^22^, ^23^ ^]^ Intravenous delivery of tumor‐targeted SAgs has been shown to facilitate the development of long‐lasting antitumor memory responses^[^ ^23^ ^]^ The broad‐spectrum activity of SAgs offers advantages over monovalent antibodies as they could engage with multiple immune cell types for synergistic tumor suppression^[^ ^24^ ^]^ Despite the potential of SAgs, however, clinical trials of TTSs have been met with unsatisfactory outcomes,^[^ ^25^, ^26^, ^27^ ^]^ which may be attributed to SAgs’ comparatively low binding affinity toward immunoreceptors.^[^ ^16^, ^28^ ^]^ Wild‐type SAgs typically have a dissociation constant in the micromolar range with immunoreceptors, which may limit their efficacy in a therapeutic context. With the aim of bolstering SAg's immune‐stimulatory activities and NK and T cell activation, directed evolution using yeast surface display and tri‐functional fusion protein designs are herein employed for multivalent ICE preparation.

To identify high‐affinity SAgs, we adopt a yeast‐based library and yeast surface display system for directed evolution^[^ ^29^ ^]^ of Staphylococcal enterotoxin B (SEB). High‐affinity SEB variants are assessed for their ligand binding affinities, and immune‐stimulatory activities, and subsequently incorporated into the design of a tumor‐targeted ICE herein termed Superantigen‐based Tri‐functional Yeast‐display‐enhanced Multivalent Immune cell Engager (STYMIE). STYMIE is constructed using an Fc backbone co‐expressing tumor‐targeted anti‐mesothelin nanobody and SAg for intravenous cancer targeting. In addition, a computationally designed cytokine, neo‐2/15, which possesses a superior safety profile, structural stability, thermostability, and activity as compared to conventional IL‐2^[^ ^30^ ^]^ is rationally incorporated into STYMIE to enhance NK and T cell activation.^[^ ^31^, ^32^ ^]^ We demonstrate the superiority of STYMIE over conventional bispecific engager design toward solid tumor suppression and inducing tumor immune cell infiltrations. In addition, SEB affinity with immunoreceptors and cytokine placement on STYMIE is shown to drastically influence fusion protein performance, highlighting critical design principles for improving tumor‐targeted ICEs.

## 2 Results

### 2.1 Directed Evolution with Yeast Display Library Yields SEB Variants with Enhanced Receptor Binding Affinity

**Figure 1:**
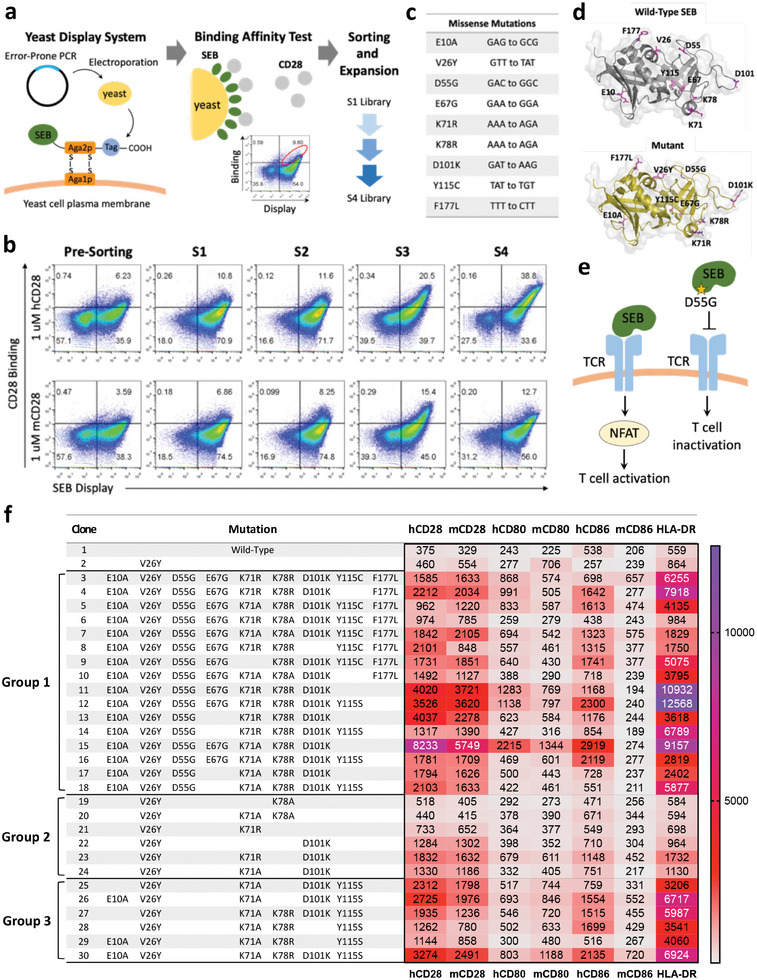
Identification of SEB variants with enhanced immunoreceptor binding affinity. a) The stepwise illustration of the SEB yeast library establishment. The library was incubated with 1 µm of recombinant human CD28, and the population exhibiting a higher binding affinity to human CD28 was sorted through flow cytometry. The sorted library was then amplified, resulting in the production of 4 subsequent generations of libraries. b) The binding affinity of all the sorted SEB libraries to mouse and human CD28 as measured by flow cytometry. c) Missense mutations were identified from 9 single clones selected from the S4 SEB yeast library. d) In the SEB structure (pdb: 1sbb), the wild‐type SEB (upper panel) and the 9 mutated amino acids on the S4 clone (SEB #3) (lower panel) are shown in the pink sticks using PyMol. e) Schematic illustration demonstrating the potential hindrance effect of the D55G mutation on TCR interaction and downstream NFAT signaling. f) The binding affinity of all the SEB variants toward the immunoreceptors (CD28, CD80, CD86, and MHC‐II) was tested using flow cytometry. The mean fluorescence of protein binding on the surface‐displayed population is shown.

Previous studies demonstrated that the binding affinity and biological functions of SAgs toward T cells can be altered by mutations in the SEB^[^ ^28^ ^]^ For example, SEB^T150A/K152A^ increased the binding affinity to CD28, although such mutation led to reduced cytokine induction in human PBMCs due to its inability to bind to CD86^[^ ^16^ ^]^ To improve the T cell‐activating ability of SEB and the binding affinity to immunoreceptors, we generated a SEB yeast library using error‐prone PCR with SEB^V26Y^ as the PCR template. As CD28 is considered a primary co‐stimulatory receptor for SAg‐induced T‐cell activation, the SEB yeast library was incubated with human CD28 recombinant proteins and sorted for strong binders via flow cytometry. After four rounds of selection, the culture was enriched with human CD28 binders (**Figure** **1**a), and the sorted libraries exhibited increased binding affinity to both mouse and human CD28 (Figure 1b). Subsequently, we selected 9 yeast single clones from the S4 library for Sanger sequencing. These 9 clones shared largely identical sequences with 9 distinctive missense mutations (Figure 1c). In the SEB structure (pdb: 1sbb), the 9 mutated amino acids in the wild‐type SEB (upper panel) and the S4 clone (lower panel) were visualized as pink sticks using PyMol (Figure 1d).

In order to evaluate the potential impact of these 9 mutations, we examined how these mutations may affect the interfaces involved in protein‐protein interactions with immunoreceptors. Among the 9 mutations, we speculated that E67G and Y115C may affect the SEB binding to MHC‐II because they abolish an existing salt bridge and a hydrogen bond from the wild‐type SEB, respectively (Figure S1a, Supporting Information). As to the TCR interaction, F177L may attenuate the binding of SEB to mouse TCR by eliminating the π‐π interaction with H47 of TCR (Figure S1b, Supporting Information). Additionally, as the D55G mutation is located near the TCR binding site for SEB (Figure S1c, Supporting Information), we speculate that the mutation may affect the interaction with TCR, thus potentially preventing the activation of downstream NFAT signaling for T cell activation (Figure 1e). On the other hand, the D101K mutation reverses the amino acid charge from negative to positive, and it creates a positive charge patch together with K71R and K78R on the same side of SEB (Figure S1d, Supporting Information). We generated three groups of SEB variants based on these amino acid alterations. The groups were categorized based on their structural similarities: group 1 includes mutations in the SEB‐TCR interfaces (D55G, F177L), the positive‐charge patch (K71R, K78R, D101K), and other mutations; group 2 only includes mutations in the positive‐charge patch; and group 3 includes mutations in the positive‐charge patch and other sites, excluding the SEB‐TCR interface mutations (D55G, F177L). We then evaluated the binding affinity between these SEB variants and the receptors CD28, CD80, CD86, and HLA‐DR using the yeast display system. Nearly all the SEB variants exhibited improved binding to all four receptors compared to SEB #1 and #2 (Figure 1f), yielding multiple SAg candidates for subsequent evaluation and STYMIE construction.

### 2.2 SEB Binds to Human CD2/CD58 and Mouse CD2/CD48 to Activate T Cells

**Figure 2:**
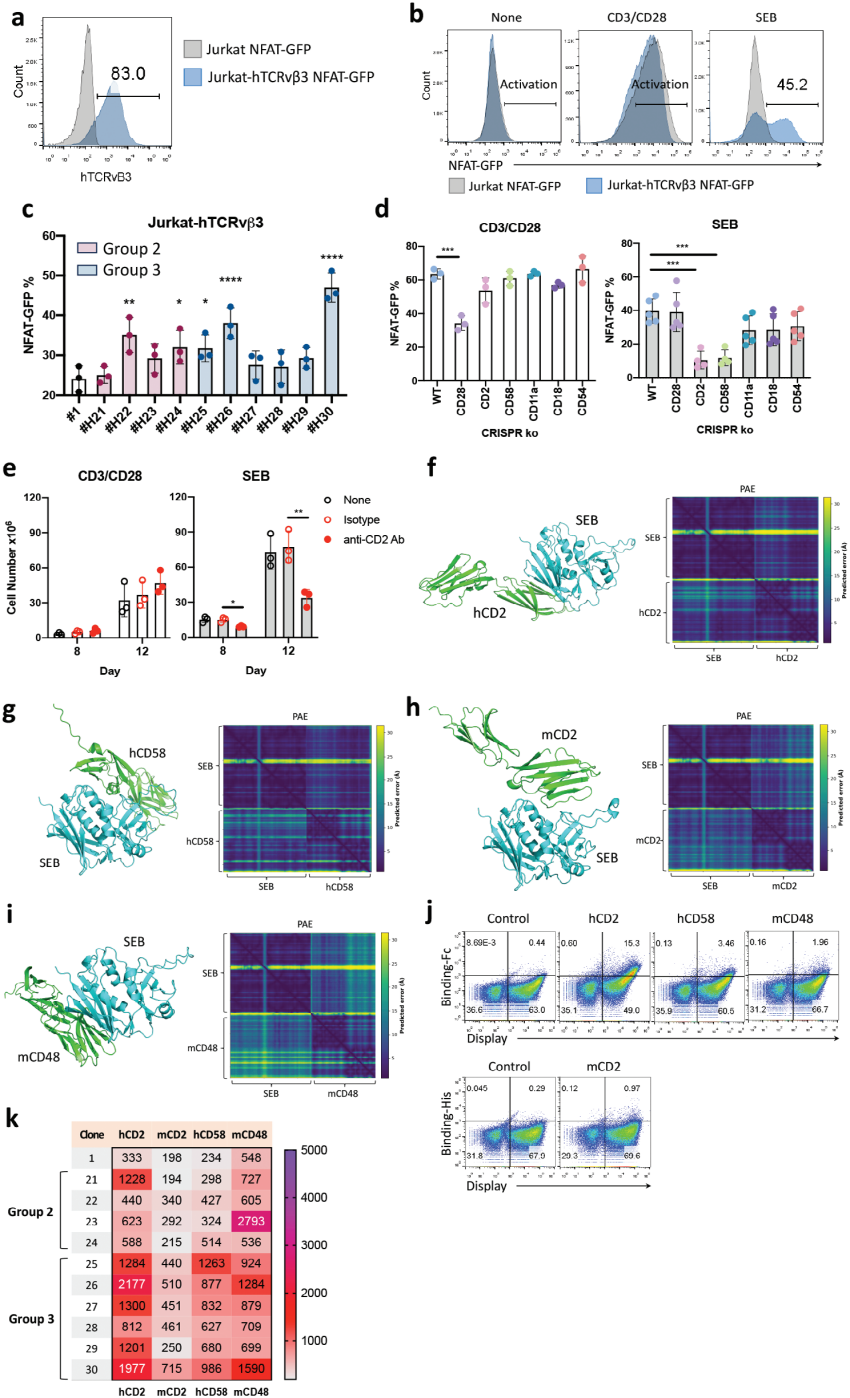
SEB binds to human CD2/CD58 and mouse CD2/CD48 to activate T cells. a) The expression level of hTCRvβ3 in the lentivirus‐transduced Jurkat NFAT‐GFP reporter cells. b) Activation of parental Jurkat NFAT‐GFP reporter cells and Jurkat‐hTCRvβ3 NFAT‐GFP reporter cells treated with anti‐CD3/CD28 antibody or 1 µg ml^−1^ of recombinant wild‐type SEB for 24 hrs. c) Activation of Jurkat‐hTCRvβ3 NFAT‐GFP reporter cells with 0.3 µg ml^−1^ recombinant SEB variants for 24 hrs. (n = 3) d) Activation of Jurkat‐hTCRvβ3 NFAT‐GFP reporter cells and other CRISPR knockout cells following treatment with anti‐CD3/CD28 or 1 µg ml^−1^ of recombinant wild‐type SEB for 24 hrs. (n = 3 to 5) e) Human PBMC proliferation by SEB upon anti‐CD2 blocking. Human PBMCs were pre‐treated with 1 µg ml^−1^ of an isotype control antibody or anti‐CD2 antibody and then treated with anti‐CD3/CD28 antibody or 1 ng ml^−1^ of recombinant wild‐type SEB for evaluation. (n = 3) f–i) Protein‐protein interactions between SEB and CD2, human CD58, and mouse CD48 as predicted with AlphaFold2‐Multimer. The ranked 0 and the predicted aligned error (PAE) score for the model ranked 0 are shown. j,k) The binding of wild‐type SEB and SEB variants toward yeast cells displaying CD2, human CD58, or mouse CD48. The mean fluorescence of protein binding on the surface‐displayed population is shown. The error bars represent mean ± SD. Statistical analyses were performed by One‐way ANOVA c) followed by the Fisher's LSD test, d) with Dunnett correction, and (e) unpaired Student's *t*‐test. (^*^ *p* < 0.05, ^**^ *p* < 0.01, ^***^ *p* < 0.001, ^****^ *p* < 0.0001).

To investigate T cell activation by the SEB variants and to explore the underlying molecular mechanism, we established a Jurkat NFAT‐GFP reporter cell line expressing human TCR Vβ3 (Jurkat‐hTCRvβ3 NFAT‐GFP), which is known to interact with SEB^[^ ^33^ ^]^ (**Figure** **2**a). Wild‐type SEB is shown to specifically activate Jurkat‐hTCRvβ3 NFAT‐GFP cells, thus validating the cell as a viable tool for examining SEB‐mediated immune cell activation (Figure 2b). Based on the Jurkat‐hTCRvβ3 NFAT‐GFP reporter cells, we observed the D55G mutation in the group 1 SEB variants impaired NFAT signaling activation (Figure S2, Supporting Information), presumably due to inhibition of SEB binding with TCR Vβ3. On the other hand, multiple SEB variants from groups 2 and 3, including variants #H22, #H24, #H25, #H26, and #H30, showed significant enhancement of reporter cell activation (Figure 2c). As SEB‐mediated T cell activation may involve co‐receptors that have yet to be elucidated,^[^ ^20^, ^34^ ^]^ we further investigated the role of several widely expressed immunoreceptors, including CD2, CD58, CD11a, CD18, and CD54, using CRISPR knockout Jurkat‐hTCRvβ3 NFAT‐GFP cells (Figure S3, Supporting Information). The validity of the CRISPR knockout reporter cell assay was confirmed via CD3/CD28 stimulation, which revealed a decrease in NFAT signaling only in the CD28 knockout cells. Interestingly, the SEB‐induced NFAT signaling was significantly reduced in cells without CD2 and CD58, which are unexplored immunoreceptors of SEB to our knowledge (Figure 2d). As CD2 binding by SEB has broad implications in understanding SEB's cross‐reactive immunostimulatory functions given that CD2 is widely expressed on NK and iNKT cells,^[^ ^35^, ^36^ ^]^ we further performed an anti‐CD2 blocking assay. The addition of anti‐CD2 resulted in the down‐regulation of SEB‐induced PBMC proliferation (Figure 2e), which reaffirms CD2 as a ligand for SEB‐mediated immune stimulation.

To gain insight into the interaction between SEB and related co‐receptors (human and mouse CD2, human CD58, and mouse CD48, which is a homolog of human CD58^[^ ^37^ ^]^), we used AlphaFold 2‐multimer to predict their protein‐protein interaction. Among the 25 predicted results for each receptor, the protein complexes were aligned and grouped into several clusters based on their structural similarities (Figures S4–S7, Supporting Information). The predicted protein complex model revealed a direct interaction between SEB and human CD2, mouse CD2, human CD58, and mouse CD48. The PAE (predicted align errors) plot displayed minimal variations in distance between residues, indicating high accuracy in the protein folding and the predicted structure (Figure 2f–i). When wild‐type SEB was displayed on yeast, it showed direct binding to human CD2, but with low binding affinity for human CD58, mouse CD2, and mouse CD48 (Figure 2j). Conversely, multiple SEB variants displayed enhanced binding affinity for human and mouse CD2, human CD58, and mouse CD48 (Figure 2k), suggesting that the SEB variants could induce elevated activation of T cells and other immune cells through engagement with these widely expressed co‐receptors.

### 2.3 High‐Affinity SEB Variants Enhance CD2‐ and CD58‐Mediated T Cell Activation

**Figure 3:**
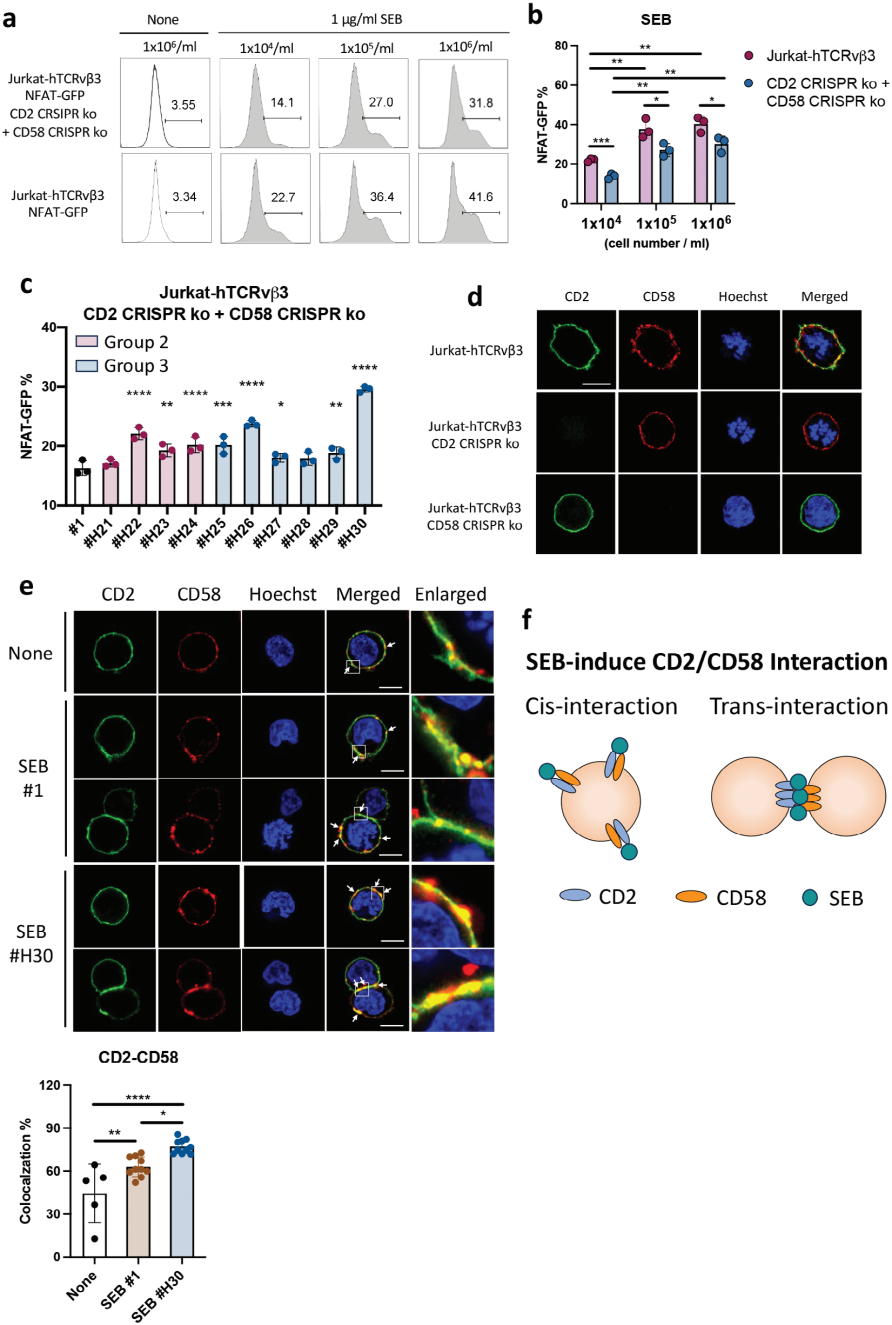
High‐affinity SEB variants enhance cis and trans‐CD2/CD58 engagement for T cell activation. a) Jurkat‐hTCRvβ3 NFAT‐GFP reporter cell activation upon 24 h treatment with 1 µg ml^−1^ recombinant SEB variants. The percentage of activated NFAT‐GFP signaling was determined using flow cytometry. b) NFAT‐GFP signaling from Jurkat‐hTCRvβ3 NFAT‐GFP reporter cells and from a mixture of 1:1 Jurkat‐hTCRvβ3 CD2 knockout and Jurkat‐hTCRvβ3 CD58 knockout reporter cells following 24 h treatment with 0.3 µg ml^−1^ of recombinant wild‐type SEB. (n = 3) c) NFAT‐GFP signaling from a mixture of 1:1 Jurkat‐hTCRvβ3 CD2 knockout and Jurkat‐hTCRvβ3 CD58 knockout reporter cells following 24 hrs treatment with 1 µg ml^−1^ SEB variants. (n = 3) d) Visualization of CD2 and CD58 molecules on Jurkat‐hTCRvβ3, Jurkat‐hTCRvβ3 CD2 knockout, and Jurkat‐hTCRvβ3 CD58 knockout cells. The cells were fluorescently labeled anti‐CD2 ^[^ ^41^ ^]^ and anti‐CD58 antibodies (red). Cells were analyzed with a laser‐scanning confocal microscope. Scale bars = 10 µm. e) Visualization and quantification of CD2 and CD58 colocalization on Jurkat‐hTCRvβ3 cells treated with 1 µg ml^−1^ of wild‐type SEB (#1) or high‐affinity SEB variant (#30) at 37 °C for 2 h. The cells were fixed and stained with fluorescently labeled anti‐CD2 and anti‐CD58 Abs. Cells were analyzed with a laser‐scanning confocal microscope. Scale bars = 10 µm. (n = 5 to 10) f) Schematic illustration of the cis‐ and trans‐interaction of CD2 and CD58 on T cells. The error bars represent mean ± SD. Statistical analyses were performed by (b) unpaired Student's *t*‐test, One‐way ANOVA (c) followed by the Fisher's LSD test, or (e) with Dunnett correction. (^*^ *p* < 0.05, ^**^ *p* < 0.01, ^***^ *p* < 0.001, ^****^ *p* < 0.0001).

The interaction between CD2 and its ligand CD58 is recognized as a co‐stimulatory signal for T cell activation. The prevailing viewpoint supports the idea that CD2 on T cells engages with its ligand, CD58, which is located on APCs.^[^ ^38^, ^39^ ^]^ However, it is important to note that both CD2 and CD58 are also expressed in T cells themselves. This suggests that interactions between CD2 and CD58 on T cells, independent of APCs, may be sufficient to induce NFAT signaling activation alongside TCR activation (Figure 2c,d; Figure S8, Supporting Information). To evaluate whether the SEB variants enhance NFAT‐GFP signaling through direct CD2 and CD58 interaction on T cells, we mixed the Jurkat‐hTCRvβ3 CD2 CRISPR knockout and Jurkat‐hTCRvβ3 CD58 CRISPR knockout NFAT‐GFP reporter cells together for SEB stimulation. SEB induced robust NFAT‐GFP signaling through the trans‐interaction of CD2 and CD58 between adjacent T cells (**Figure** **3**a upper panel and Figure 3b). In addition, we observed that Jurkat‐hTCRvβ3 NFAT‐GFP reporter cells exhibited stronger SEB‐induced NFAT‐GFP signaling compared to the mixture of CD2 and CD58 knockout cells, suggesting that cis CD2 and CD58 interactions may contribute to SEB‐mediated T cell activation^[^ ^40^ ^]^ (Figure 3a lower panel and Figure 3b). Upon mixing Jurkat‐hTCRvβ3 CD2 knockout and Jurkat‐hTCRvβ3 CD58 knockout reporter cells with SEB variants, variants such as #H22, #H23, #H24, #H25, #H26, #H27, #H29, and #H30 significantly enhanced NFAT signaling activity compared to SEB #1 (Figure 3c). Further analysis by fluorescence microscopy showed that SEB induces colocalization of CD2 and CD58 on Jurkat‐hTCRvβ3 cells through both cis‐ and trans‐interactions. Notably, SEB variant #H30, which induces the strongest NFAT signaling activation, increased the colocalization percentage of CD2 and CD58 compared to the wild‐type SEB (Figure 3d,e). These results indicate that SEB binds to both CD2 and CD58 molecules, leading to an enhanced NFAT signaling pathway through both cis‐ and trans‐interaction of CD2 and CD58 (Figure 3f). In addition, high‐affinity SEB variants capable of enhancing T cell activation through improved CD2 and CD58 bridging were identified.

### 2.4 High‐Affinity SEB Variants Enhanced T Cell and NK Cell Activation

**Figure 4:**
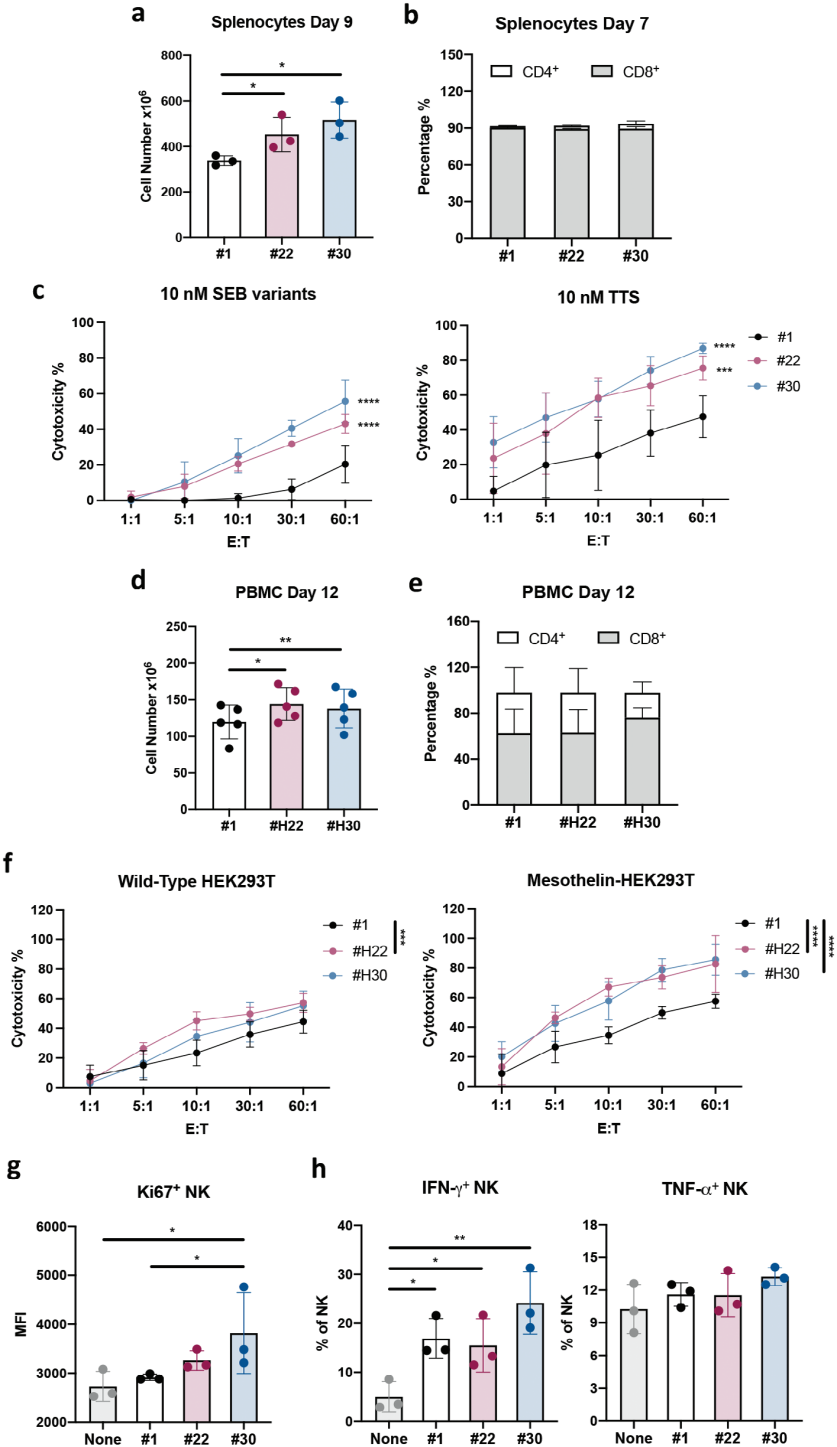
Characterization of the SEB‐induced T cell proliferation, T cell‐mediated cytotoxicity effects, and NK cell activation. a) Cell proliferation and b) CD4 to CD8 ratio following mouse splenocytes stimulation with 0.1 µg ml^−1^ of purified SEB variants supplemented with Neo‐2/15. (n = 3) c) Cytotoxicity assay using SEB variants‐expanded splenocytes at different E:T ratios as effector cells. The splenocytes were amplified by 1 µg ml^−1^ of the recombinant SEB variants for 5 days and co‐cultured with the mesothelin‐CT26 in the presence of 10 nM of recombinant SEB variants (left) or recombinant tumor‐targeted SEB variants (right). (n = 3) d) Cell proliferation and e) CD4 to CD8 T cell ratio following human PBMCs stimulation with 1 ng ml^−1^ of purified SEB variants supplemented with Neo‐2/15. (n = 5) f) Cytolytic of wild‐type HEK239T and mesothelin‐HEK293T cells upon co‐incubation with SEB‐variant‐amplified human PBMCs and 10 nM recombinant tumor‐targeted SEB variants. (n = 3 to 5) g,h) Splenocytes were stimulated with 1 µg ml^−1^ of purified SEB variants supplemented with 10 ng ml^−1^ Neo‐2/15. After 48 hrs, the proliferation marker ki67 and the cytokines secretion level of NK cells were measured using flow cytometry. (n = 3) The error bars represent mean ± SD. Statistical analyses were performed by (a, d) paired Student's *t*‐test and (g, h) One‐way ANOVA followed by the Fisher's LSD test. (^*^ *p* < 0.05, ^**^ *p* < 0.01). (c, f) Data was analyzed by Two‐way ANOVA with Tukey correction. (^***^ *p* < 0.001, ^****^ *p* < 0.0001).

To investigate the biological functions of high‐affinity SEB variants in inducing T cell proliferation, activation, and cytolysis against tumors, we selected the variants with the highest capability to induce NFAT signaling from each group, namely SEB #22 and #30. Splenocyte stimulation with purified SEB variants revealed that SEB #22 and #30 significantly increased cell proliferation compared to SEB #1, starting from 1.8 × 10^6^ cells at the initiation of stimulation to ≈500×10^6^‐600 × 10^6^ cells by day 9 post‐stimulation (**Figure** **4**a). This amplification was greater than that observed with wild‐type SEB #1. (Figure 4a). Flow cytometry analysis of the expanded cell populations revealed that T cells comprised 30% of splenocytes on day 0 (data not shown), increasing to 90–95% of the total splenocyte population by day 7, with CD8 T cells representing 85–95% of the SEB‐amplified splenocyte population (Figure 4b). To assess SEB‐induced T cell cytotoxicity through tumor‐targeted engager design, we engineered a tumor‐targeted superantigen by fusing the SEB variants with an anti‐mesothelin nanobody^[^ ^42^ ^]^ Cytolytic studies demonstrated that T cells activated by the SEB variants #22 and #30 displayed stronger T cell cytotoxicity against cancer cells as compared to SEB #1. In addition, co‐incubation with the tumor‐targeted SEBs resulted in an overall enhancement to T cell‐mediated cytolysis, indicating effective bispecific engagement by the superantigen‐based engager (Figure 4c). Examination of SEB activities with human PBMCs showed comparable results as with mouse splenocytes. SEB #H22 and #H30 triggered higher rates of human T cell proliferation compared to SEB #1, starting from an initial cell count of 1 × 10^6^ (Figure 4d). Total T cells constituted ≈95% of the total PBMC population, with CD8 T cells representing ≈60–85% of the cultured PBMCs (Figure 4e). SEB #H22 and #H30‐amplified human PBMCs also demonstrated stronger T cell cytotoxicity in the presence of tumor‐targeted superantigen engager against mesothelin‐expressing HEK293T cells (Figure 4f). As SEB #22 and #30 exhibit enhanced affinity to CD2, which is a putative NK cell ligand associated with immunological synapse formation, activation, and cytotoxic activity^[^ ^43^ ^]^ we further assessed NK activation by these SEB variants. Mouse splenocyte stimulation with purified SEB variants showed that their affinity enhancement elevated SEB's NK‐activating capacity, with SEB #30 inducing significantly higher NK cell proliferation signaling as compared to SEB #1 (Figure 4g). Additionally, all SEB variants resulted in higher levels of IFN‐γ secretion compared to the non‐stimulated group, with SEB #30 exhibiting a marginal IFN‐γ enhancement compared to SEB #1 (Figure 4h). Comparable TNF‐α levels were observed among the different SEB‐stimulated NK cells. These results validate the enhanced T cell and NK cell stimulating activity of high‐affinity SEB variants.

### 2.5 Rational Integration of Neo‐2/15 in STYMIE Enhances T Cell and NK Cell Expansion

**Figure 5:**
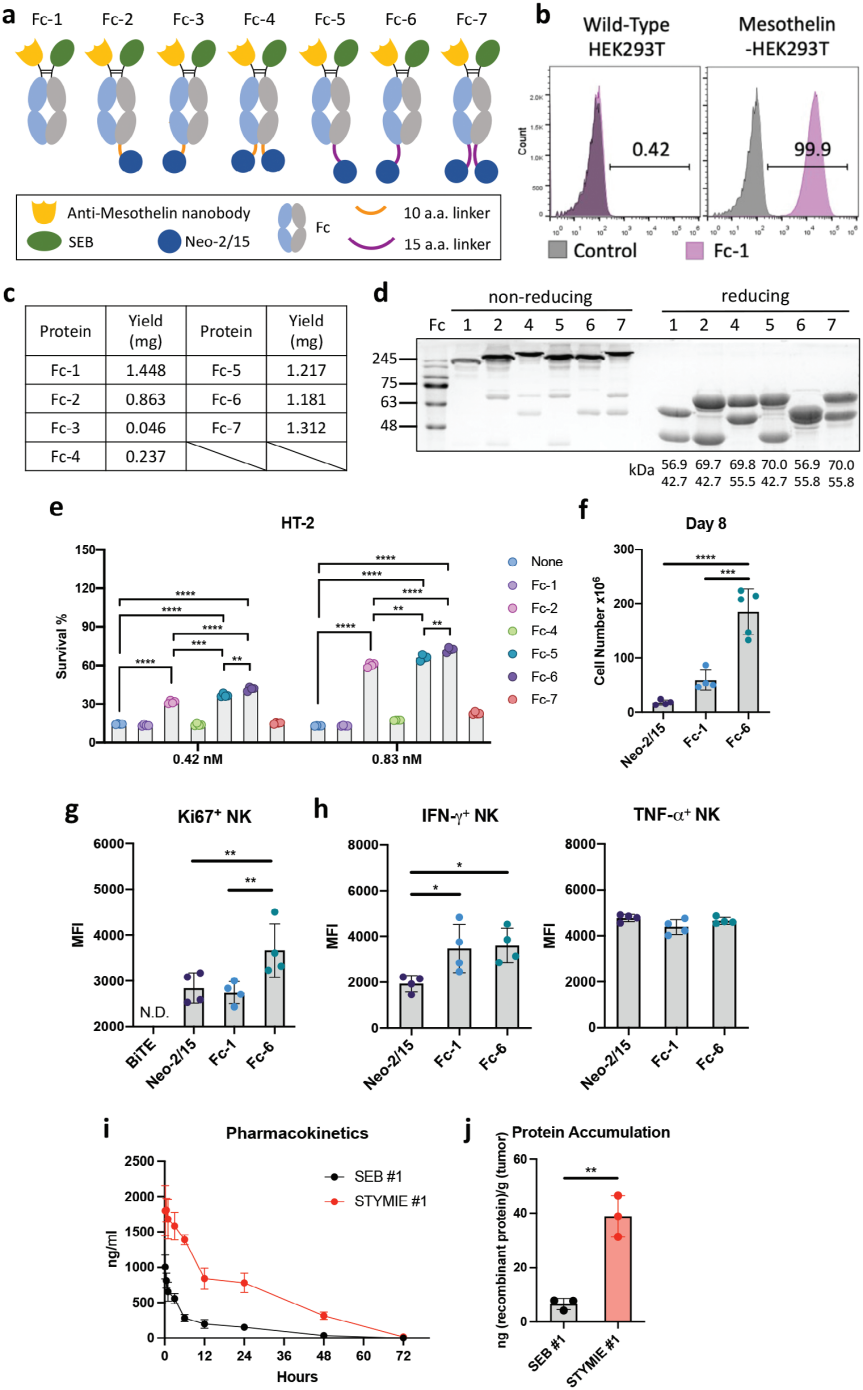
Design and functional characterization of Neo‐2/15‐functionalized STYMIE. a) Schematic illustration of STYMIE fusion protein designs with varying Neo‐2/15 functionalization. b) Mesothelin‐specific cancer targeting by the STYMIE construct as confirmed by flow cytometric analysis. c) Protein production yield for each purified STYMIE from a 30 ml culture of expiCHO cells. d) The heterodimer structures of the STYMIE proteins were examined under non‐reduced or reduced conditions using SDS‐PAGE. e) HT‐2 proliferation assay following cellular treatment with Neo‐2/15 or recombinant STYMIE. The viability of the HT‐2 cells was measured following 3 days of proliferation using a CCK‐8 assay. (n = 4) f) Mouse splenocytes proliferation upon incubation with 3 nM of recombinant Neo‐2/15, Fc‐1 or Fc‐6 proteins. The cells were subcultured every 3 days and supplemented with fresh STYMIE recombinant proteins. Cell numbers were counted on day 8. (n = 4 to 5) g) Proliferative activity and h) cytokine secretion by NK cells upon splenocyte stimulation with 3 nM of recombinant BiTE, Neo‐2/15, Fc‐1, or Fc‐6 protein for 48 hrs. (n = 4) i) The pharmacokinetics of SEB #1 and STYMIE #1 in vivo. (n = 3) j) Accumulations of SEB #1 and STYMIE #1 in the meso‐CT26 tumor‐bearing mice. (n = 3) The error bars represent mean ± SD. Statistical analyses were performed by (e) unpaired Student's *t*‐test, (f) One‐way ANOVA with Tukey correction, and (g, h) One‐way ANOVA followed by the Fisher's LSD test. (^*^ *p* < 0.05, ^**^ *p* < 0.01, ^***^ *p* < 0.001, ^****^ *p* < 0.0001).

Recent development of ICEs has adopted cytokine integration into fusion protein designs for enhancing anticancer immune responses.^[^ ^44^, ^45^ ^]^ This approach takes advantage of the immune stimulatory functions of cytokines, enabling cytokine therapeutics to specifically tackle the challenges associated with cancer therapy.^[^ ^46^, ^47^ ^]^ However, cytokine placement in the fusion protein structure may influence its immune stimulatory functionality, and its effect on ICE functionality has been seldom explored. To investigate if cytokine positioning may influence ICE performance, we created variants of triple fusion (TF) proteins comprising anti‐mesothelin nanobody, SEB variants, and a computationally designed cytokine Neo‐2/15^[^ ^30^ ^]^ in three different structural arrangements (Figure S9a, Supporting Information). The three TF proteins, designated as TF‐1, TF‐2, and TF‐3, contain Neo‐2/15 at the C‐terminus position, in between nanobody and superantigen, or at the N‐terminus position, respectively. Splenocyte proliferation study revealed synergistic cellular expansion with the fusion protein designs, and cytokine positioning was found to strongly influence the performance of the fusion proteins (Figure S9b, Supporting Information). The finding of positional variation in cytokine functionality prompted us to conduct a comprehensive design analysis for cytokine incorporation in STYMIE development, which adopts a knobs‐into‐holes heterodimer IgG1 Fc design for circulatory half‐life extension. Using wild‐type SEB, multiple STYMIE variations possessing Neo‐2/15 with different cytokine positioning and linker length were prepared for the identification of optimal STYMIE design (**Figure** **5**a). We first confirmed the mesothelin‐specific cancer‐targeting by the STYMIE backbone through protein binding analysis with wild‐type HEK293T and mesothelin‐HEK293T cells (Figure 5b). STYMIE with varying Neo‐2/15 functionalization were then prepared, and all STYMIE variants with the exception of Fc‐3 protein were readily derived with high protein yield (Figure 5c). The low yield of Fc‐3 may be attributed to limited flexibility between Neo‐2/15 and Fc domain, which could impede the pairing of knob‐Fc and hole‐Fc into a complete Fc domain. Analyses by SDS‐PAGE and FPLC validated that the purified STYMIE proteins possessed properly dimerized Fc structure (Figure 5d; Figure S10, Supporting Information). Upon examining the influence of Neo‐2/15 functionalization on STYMIE for stimulating HT‐2 cell expansion, we observed that Fc‐4 and Fc‐7, which possess bivalent Neo‐2/15 functionalization, showed minimal HT‐2 stimulation (Figure 5e). The low immune stimulatory activity of Fc‐4 and Fc‐7 can be attributed to steric shielding that limits cytokine engagement with their putative ligand. In contrast, STYMIE with monovalent Neo‐2/15 (Fc‐2, Fc‐5, and Fc‐6), showed robust HT‐2 proliferation. As Fc‐6 induced the highest HT‐2 proliferation, it was selected as the design of choice for subsequent characterization and STYMIE construction. In a mouse splenocyte proliferation study, Fc‐6 resulted in a significantly higher proliferation rate as compared to Neo‐2/15 and Fc‐1, thus affirming the combinatorial activity of the tri‐functional engager (Figure 5f). Evaluation of NK cells from the proliferated splenocytes also showed that Fc‐6 induced higher proliferative activity among the NK cells (Figure 5g). In contrast, a conventional BiTE comprised of anti‐mesothelin nanobody and anti‐CD3 ScFv failed to support the survival of NK cells in the splenocytes. Examination of cytokine secretion among the NK cells also showed that Fc‐1 and Fc‐6 induced stronger IFN‐γ secretion in comparison to the Neo‐2/15 group, although all three groups resulted in comparable TNF‐α secretion (Figure 5h). These results reaffirm the multivalent immune cell engagement of STYMIE for T cell and NK activation. In addition, an optimal STYMIE structure that maximizes cytokine functionality was identified. To assess whether the design of STYMIE resulted in prolonged pharmacokinetics in vivo when compared to the SEB, fluorescence‐labeled SEB #1 and STYMIE #1 (STYMIE containing wild‐type SEB) were intravenously administered into BALB/c mice. Serum samples were collected starting from 10 min post‐injection and evaluated at various time points to measure fluorescence levels, enabling the determination of the pharmacokinetic profiles of SEB #1 and STYMIE #1. The findings revealed that STYMIE #1 demonstrated an extended duration in circulation within the body compared to SEB #1 (Figure 5i). Upon injection of fluorescence‐labeled SEB #1 and STYMIE #1 into mesothelin‐CT26 tumor‐bearing mice, we observed elevated accumulation of STYMIE #1 in tumors, highlighting its tumor‐targeting characteristic for anticancer therapy (Figure 5j).

### 2.6 Intravenous Administration of STYMIE with High‐Affinity Superantigen Variants Induces Multivalent Tumor Lymphocyte Infiltration and Anti‐Tumor Activity

**Figure 6:**
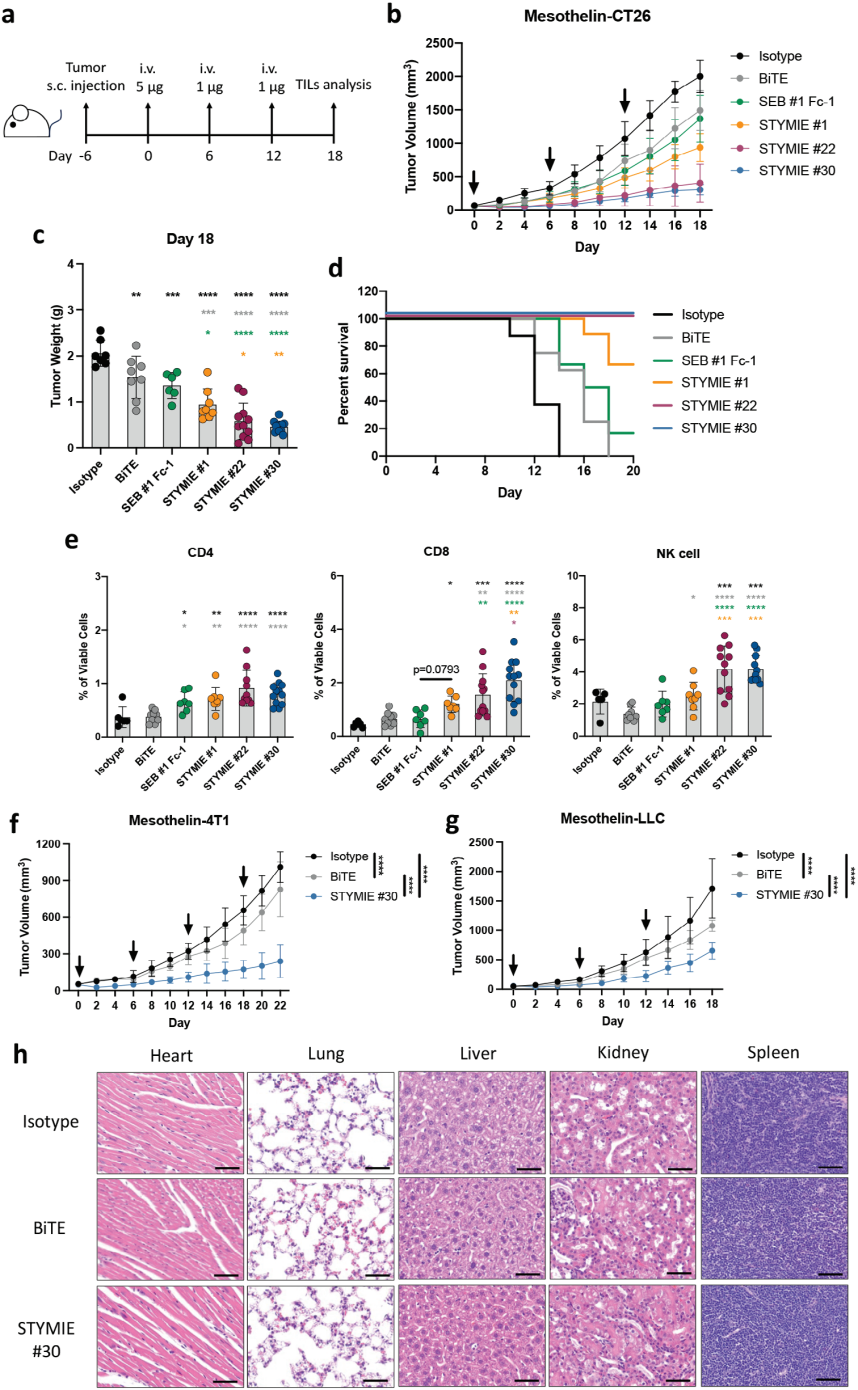
STYMIE induces robust anti‐tumor activity and tumor lymphocyte infiltration. a) Treatment and analysis schedule for mouse models of CT26 colon cancer, 4T1 breast cancer, and Lewis lung carcinoma. b) The tumor growth curve and c) the tumor weight of the mesothelin‐CT26 tumor‐bearing mice in the different treatment groups. (n = 6 to 12) d) The Kaplan‐Meier analysis for different treatment groups. The mouse was considered dead when the tumor volume exceeded 1000 mm^3^. (n = 6 to 12) e) TILs analysis of excised tumors from each treatment group. The levels of CD4 T cells (CD45^+^CD4^+^), CD8 T cells (CD45^+^CD8^+^), and NK cells (CD45^+^CD335^+^) were determined via flow cytometry. (n = 5 to 11) f) 4T1 breast cancer and (n = 6 to 8) g) Lewis lung carcinoma growth kinetics following treatment with STYMIE #30. (n = 5 to 7) h) Representative H&E stains of heart, lung, liver, kidney, and spleen of mice treated with isotype Fc, BiTE, or STYMIE #30. Scale bars = 50 µm. The error bars represent mean ± SD. Statistical analyses were performed by (c, e) One‐way ANOVA followed by the Fisher's LSD test and (f, g) Two‐way ANOVA with Tukey correction. The black asterisk represents the statistical results compared to the isotype, the gray asterisk represents the results compared to BiTE, the green asterisk represents the results compared to SEB #1 Fc‐1, and the orange asterisk represents the results compared to STYMIE #1. (^*^ *p* < 0.05, ^**^ *p* < 0.01, ^***^ *p* < 0.001, ^****^ *p* < 0.0001).

To evaluate how superantigen affinity would influence the therapeutic efficacy of ICEs, we prepared STYMIE with wild‐type SEB, SEB #22, or SEB #30, herein designated as STYMIE #1, #22, and #33, respectively. The purified STYMIE were intravenously administered into mesothelin‐CT26 tumor‐bearing mice. For comparison, a mesothelin/CD3‐targeted BiTE^[^ ^48^ ^]^ and a non‐Neo‐2/15‐functionalized Fc‐1 construct expressing mesothelin‐targeted nanobody and SEB #1 (SEB #1 Fc‐1) were administered (**Figure** **6**a). All ICE treatments resulted in tumor growth inhibition, with SEB #1 Fc‐1 exhibiting marginal improvement over BiTE in reducing tumor growth and extending mice survival (Figure 6b–d; Figure S11, Supporting Information). Neo‐2/15 functionalization in STYMIE was shown to enhance the anticancer effect. In particular, STYMIE #22 and #30 induced significantly better tumor inhibition and conferred survival benefits as compared to STYMIE #1. To confirm that the improved efficacy of the STYMIE variant is attributable to enhanced superantigen affinity to immunoreceptors, we examined SEB #1 and #30 under surface plasmon resonance spectroscopy. CD28 showed a binding affinity of 4.848 µM against SEB #30, whereas the binding affinity to SEB #1 exceeded the measurable range, thus correlating the therapeutic advantages of SEB to immunoreceptor affinity enhancement from directed evolution (Figure S12, Supporting Information). To demonstrate the ability of STYMIE to recruit both NK cells and T cells to the tumor microenvironment, tumor‐infiltrating leukocyte analysis was performed on day 18 following the initial treatment. Treatment with STYMIE #22 and #30 resulted in the highest increase in tumor‐infiltrating CD4^+^ T cells, CD8^+^ T cells, and NK cells (Figure 6e), whereas neutrophils, macrophages, and B cells remained largely unaffected across all treatment and control groups (Figure S13, Supporting Information). Notably, the mice did not experience significant body weight loss during the treatment regimen (Figure S14, Supporting Information), supporting the safety and clinical relevance of intravenous STYMIE delivery.

Given the exceptional immune‐stimulatory activity and anticancer effect of STYMIE #30, we further assessed its treatment efficacy against a 4T1 breast cancer model^[^ ^49^ ^]^ and a Lewis lung carcinoma (LLC) model^[^ ^50^ ^]^ Mesothelin‐expressing 4T1 and LLC cell lines (mesothelin‐4T1 and mesothelin‐LLC) were first established and injected subcutaneously into BALB/c mice and C57BL/6 mice, respectively. 5 µg of STYMIE and a BiTE control were given when the tumor volume reached ≈50–100 mm^3^, followed by injections of 1 µg protein every 6 days. In both the 4T1 (Figure 6f) and LLC tumor models (Figure 6g), STYMIE #30 exhibited significant tumor inhibition as compared to both the control group and the BiTE group (Figure 6f–g; Figure S15, Supporting Information). To further assess the in vivo safety of STYMIE, we examined tissue histology of the heart, lung, liver, kidney, and spleen in addition to body weight monitoring (Figure S16, Supporting Information). Two days following the second STYMIE injection, no structural alterations or immune cell infiltration were observed in the examined organs (Figure 6h) and no hematological adverse events were observed (Table S1, Supporting Information), highlighting STYMIE as a viable ICE design for intravenous anticancer treatment against various types of solid tumors.

## 3 Discussion

While conventional Immune Cell Engagers (ICEs) have demonstrated promise in treating hematologic malignancies,^[^ ^1^, ^2^ ^]^ they still grapple with limitations that necessitate improvement. Typically, conventional ICEs, particularly TCEs, rely solely on the anti‐CD3 arm without integrating a T cell co‐stimulatory signaling arm, which is pivotal for enhancing T cell activation^[^ ^51^ ^]^ Moreover, TCEs exhibit a limited impact on innate immune cells and encounter challenges in inducing durable immune memory. Cold tumors, characterized by sparse T cell infiltration and cytokine production, pose obstacles to effective antitumor immunity by ICEs^[^ ^52^ ^]^ In this study, we introduce the development of a novel T cell engager named STYMIE, which embodies a multifunctional design aimed at augmenting T cell activation and expansion. STYMIE presents a promising avenue for cancer immunotherapy, as it addresses several limitations of conventional ICEs by employing superantigens to engage multiple immune cells, evolving SEB to efficiently enhance their ability to activate both T cells and NK cells, and incorporating cytokines to support long‐term T cell survival and enhance therapeutic efficacy. Results from i*n vivo* experiments underscore the robust anti‐tumor effects of STYMIE, leading to increased survival rates and alterations in the tumor microenvironment through enhanced immune cell infiltration. Crucially, we demonstrated the safety of STYMIE in in vivo applications, as it did not result in significant adverse effects or structural alterations in vital organs. These findings collectively bolster the potential of STYMIE as a potent tool in cancer immunotherapy, with broad implications for future research and development.

Recent studies have revealed that the activation of both T cells and NK cells can synergistically enhance the anti‐tumor effect^[^ ^13^ ^]^ which provides collaborative actions as NK cells can maintain their anti‐tumor efficacy against MHC class I‐deficient tumors that are resistant to cytotoxic T cells. In addition, the recruitment of NK cells has been shown to elevate antigen‐specific CD8 T cell induction and cytotoxic T cell fitness,^[^ ^53^, ^54^, ^55^ ^]^ thus justifying the design of multivalent ICEs for simultaneous T cell and NK cell activation. Here, we leveraged high‐affinity superantigens for the development of STYMIE, which inhibits tumor growth through engagement with both T cells and NK cells. Through directed evolution, we identified multiple SEB variants with enhanced binding affinity to immunoreceptors and improved T cell and NK cell activation capacity. This proves to be a critical step for improving the efficacy of SAg‐based ICE. Our findings indicate that the SEB variants not only exhibited improved binding affinity to CD28 but also showed an overall enhancement in binding affinity to other immunoreceptors (Figures 1f and 3k). Notably, we made the observation that the D55G mutation located near the TCR interacting interface on SEB impaired NFAT signaling activation as high‐affinity SEBs with the mutation failed to stimulate reporter cells (Figure S2, Supporting Information). The finding offers insights toward future superantigen engineering, which should avoid mutations at the TCR contact sites.

In addition to the enhanced immune activation provided by the engineered SEB, we highlight the potential of cytokine supplementation in STYMIE to support the long‐term survival of immune cells and enhance therapeutic effectiveness. Stimulating splenocytes with SEB together with Neo‐2/15 resulted in a combinatorial effect in inducing significant expansion of T cells (Figure 5f) and up‐regulated proliferation signals of NK cells in the splenocytes (Figure 5g). To incorporate all proteins into the STYMIE construct, we designed different protein module configurations as they could potentially impact the functionality and stability of each protein. Our results demonstrate that tethering Neo‐2/15 to its N‐terminus while leaving its C‐terminus untethered leads to optimal functionality. Additionally, steric considerations associated with linker length and cytokine positioning also prove to be important factors for designing antibody‐cytokine fusion proteins.

Mechanistically, we uncovered that SEB activates T cells through the intricate interplay of CD2 and CD58 in both cis‐ and trans‐interactions. Moreover, we observed that SEB variants exhibit the capacity to enhance T cell activation via CD2 and CD58 interactions. In our binding affinity tests (Figure 2k), we found that SEB #30 has a higher binding affinity toward CD2, CD58, and CD48 compared to SEB #1. This increased binding affinity of SEB #30 explains why it enhances the NFAT signaling pathway through CD2 and CD58. This result aligns well with the functional analysis and animal studies. It is noteworthy that a substantial proportion of adult CD8 T cells lack the CD28 co‐stimulatory molecule, and the gradual expansion of this T cell subset is a hallmark of immune system aging.^[^ ^56^, ^57^ ^]^ The loss of CD28, a critical costimulatory molecule, is associated with tumor cell resistance to immunotherapeutic interventions^[^ ^58^ ^]^ Additionally, heterogeneous populations of CD8^+^CD28^−^ senescent T cells have been identified in various solid and hematogenous tumors. Recent research has elucidated the significance of the CD2/CD58 as a main co‐stimulatory pathway in human CD28^−^CD8^+^ T cells^[^ ^59^ ^]^ This discovery implies that SEB variants, which enhance co‐stimulatory signals through CD2 and CD58 interactions, may hold the potential to rejuvenate T cell responses, thereby offering new opportunities for cancer immunotherapy strategies.

In our animal model experiments, we observed the promising therapeutic efficacy of STYMIE in mouse models of colorectal cancer, breast cancer, and lung cancer, providing compelling evidence for its potential in cancer immunotherapy (Figure 6d,h,i). Furthermore, specific variants of STYMIE, such as STYMIE #30, demonstrated prominent tumor inhibition compared to STYMIE #1, emphasizing the critical role of engineered SEB variants in enhancing immune cell activation for combating tumors. Importantly, we observed that a higher density of TILs during treatment correlated with improved treatment outcomes. This was supported by the results indicating increased infiltration of various immune cells, including CD4 T cells, CD8 T cells, and NK cells, in the STYMIE #30 groups (Figure 6g). These results highlight engineering advances in ICE and tumor‐targeted superantigen (TTS) therapies toward addressing the immune‐suppressive microenvironment of solid tumors.

## 4 Experimental Section

### Yeast Surface Display Library Construction

Yeast expression vectors were propagated in the *E. coli* strain XL‐10Gold. To prepare the SEB library by directed evolution, random mutagenesis was performed by 20 cycles of the SEB DNA template PCR amplification supplemented with 1.5 µm 8‐oxo‐dGTP (Jena Bioscience, NU‐1117L) and dPTP (Jena Bioscience, NU‐1119L). To bridge the SEB PCR product and the yeast expression vector, the second round of PCR was conducted to extend the overlapping region (50 bp) at the end of the SEB PCR product to enable the in vivo recombination in the yeast cells. 5 ug of purified SEB PCR products and 1 ug of linearized yeast expression vector were then pelleted with the PelletPaint (Merck Millipore, 69049) according to the manufacturer's protocol. The yeast cells strain EBY100 was freshly inoculated to the YPD plate and a single colony was grown in the YPD medium to the log phase (absorbance of 1.3–1.5 at O.D. 600 nm). Tris‐DTT buffer (100 mm Tris HCl, 10 mm dithiothreitol, pH 9.4) was added to the yeast cell culture and incubated for 20 min. Yeast cell culture was pelleted and resuspended in the ice‐cold E buffer (10 mm Tris, 270 mm sucrose, 1 mm MgCl_2_, pH 7.5) and the PelletPaint‐precipitated DNA mixture. The yeast cells and DNA mixture were then electroporated at 0.54 kV and 25 mF without a pulse controller, followed by recovery in YPD medium at 37 °C for 60 min. The transformants were cultured in SDCAA medium for 2–3 days and passage twice to eliminate the untransformed cells.

### Yeast Library Selection

To induce the display of the SEB yeast library, the yeast cells were diluted in the SGCAA medium (2% galactose, 0.67% yeast nitrogen base, 0.5% casamino acids, 0.54% Na_2_HPO_4_, 0.86% NaH_2_PO_4_) to an absorbance of 0.8‐1 at O.D. 600 nm. Yeast cells were cultured at 30 °C for 20–24 h. Induced yeast cells were incubated with 1 µm human Fc tagged human CD28 recombinant protein (SinoBiological, 11524‐H02H) at 4 °C for 2 h. Then, yeast cells were washed with PBS once and stained with the anti‐human IgG Fc antibody (Abcam, ab131612) and the anti‐HA tag antibody (Biolegend, 682404) at 4 °C for 30 min. The yeast cells were washed with PBS twice and analyzed with flow cytometry. The top 0.7–1.5% of the double‐positive cells were retained by the Bio‐Rad Sorter and recovered by at least twice of passages to amplify the binders. Yeast cells were further sorted for another 2 rounds to obtain SEB variants with higher binding affinity to human CD28. For the other immunoreceptors binding affinity tests, induced yeast cells were incubated with 1 µm human Fc tagged mouse CD28 recombinant protein (SinoBiological, 50103‐M02H), 5 µm His tagged human CD80 recombinant protein (SinoBiological, 10698‐H08H), 5 µm His tagged mouse CD80 recombinant protein (SinoBiological, 50446‐M08H), 5 µm His tagged human CD86 recombinant protein (SinoBiological, 10699‐H08H), 5 µm His tagged mouse CD86 recombinant protein (SinoBiological, 50068‐M08H), 2 µm His tagged human HLA‐DRA recombinant protein (Cusabio, CSB‐EP360793HU), 0.5 µm human Fc tagged human CD2 recombinant protein (SinoBiological, 10982‐H02H), 2 µm His tagged mouse CD2 recombinant protein (SinoBiological, 50537‐M08H), 0.5 µm human Fc tagged human CD58 recombinant protein (SinoBiological, 12409‐H02H), and 0.5 µm human Fc tagged mouse CD48 recombinant protein (SinoBiological, 50415‐M02H) at 4 °C for 2 h. Yeast cells were washed with PBS once and stained with the anti‐human IgG Fc antibody (Abcam, ab131612), the anti‐His tag antibody (Biolegend, 362603), and the anti‐HA tag antibody (Biolegend, 682404) at 4 °C for 30 min. The yeast cells were washed with PBS twice and analyzed with flow cytometry.

### Sequencing of Clones from the Yeast Library

The last round of the sorted yeast cells from the SEB library was inoculated on the SDCAA plate and incubated at 30 °C for 3–4 days. Single colonies were picked into the SDCAA medium and incubated at 30 °C. The plasmid DNA was extracted from yeast cells using the yeast plasmid miniprep kit (ZYMO Research, D2004) according to the manufacturer's protocol.

### Generation of the Mesothelin‐Expressing Cell Lines

Mesothelin‐expressing CT26, 4T1, LLC, and HEK293T stable cell lines were established via lentiviral transduction. For lentiviral production, 1.36 µg pCMV‐dR8.91, 0.165 µg pMD2‐G, and 1.5 µg of the mesothelin expression plasmid were mixed with 30 µg/ml PEI (Sigma–Aldrich, SIG764604‐1G) in the Opti‐MEM (Gibco, 31985‐070) at room temperature for 30 min. The mixture was then added to 8×10^5^ HEK 293T cells. After 16 h, the medium was replaced, and the supernatant was harvested 72 h post‐transfection. For lentiviral transduction, cells were incubated with the virus soup in the presence of 8 µg ml^−1^ polybrene (Merck, TR‐1003‐G) for 16 h, followed by selection with puromycin (InvivoGen, ant‐pr‐1) for 10 days. The mesothelin expression levels of CT26 and HEK293T cells were confirmed by using flow cytometry.

### Fc Fusion Protein Expression in Mammalian Cells and Protein Purification

The ExpiCHO‐S cells (Gibco) were cultured in ExpiCHO expression medium (Gibco, A29100) at 37 °C under 8% CO_2_ with shaking in a 125 ml Erlenmeyer flask (Corning, 431143). To express the STYMIE, the ExpiCHO‐S cells (Gibco) were co‐transfected with the hetero Fc plasmids at a ratio of 1:1, following the manufacturer's instructions. The supernatant was harvested after 8 days and purified using the HiTrap Protein G column (Cytiva, 17040401). The protein purity was further examined and/or purified by size‐exclusion chromatography using a Superdex 200 increase column (GE Healthcare) in PBS.

### Structure Models and Protein‐Protein Interaction Analysis

Cartoon models of protein structure were created in PyMol (www.pymol.org). For the prediction of protein‐protein interactions between SEB and the receptors, the sequences of SEB, human and mouse CD2, human CD58, and mouse CD48 were retrieved from the UniProt database (https://www. uniprot.org). The protein‐protein interactions between SEB and the receptors were predicted using AlphaFold2‐Multimer (downloaded from https://github.com/deepmind/alphafold).

### Generation of CRISPR Knockout Cells

CRISPR knockout plasmids were designed to carry both the SpCas9 protein and sgRNA sequences. The sgRNA sequences were designed to target specific genes using the Broad Institute CRISPick tool (https://portals.broadinstitute.org/gppx/crispick/public), employing default parameters for the Human GRCh38 (NCBI) reference genome, along with the tracrRNA sequence from Hsu (2013). Jukrat‐hTCRvβ3 CRISPR knockout cells were generated with a Lonza 4D‐Nucleofector System (Lonza) using the program CL‐120 and the 4D‐Nucleofector kit (Lonza, V4XC‐1032). Briefly, 2×10^5^ cells were electroporated with 1 µg of CRISPR plasmids and immediately recovered in 1 ml medium at 37 °C for 3 days. Then, the cells underwent a selection process utilizing puromycin and cell sorting to isolate the knockout cells.

### TF Protein Binding Analysis

10 nM of recombinant TF proteins were co‐incubated with both wild‐type HEK293T and mesothelin‐HEK293T cells at 4 °C for 1 h. After washes, the cells were incubated with anti‐His tag antibody (Biolegend, 362603) at 4 °C for 30 min. The ability of the anti‐mesothelin nanobody to bind to mesothelin was confirmed by flow cytometry.

### Confocal Microscopy

To examine the co‐localization of human CD2 and CD58, Jukrat‐hTCRvβ3, Jukrat‐hTCRvβ3 CD2 CRISPR knockout, and Jukrat‐hTCRvβ3 CD58 CRISPR knockout cells were incubated with 1 µg/ml SEB recombinant protein at 37 °C for 2 h. Then, the cells were washed with 1 ml of 1X PBS and fixed with 1 ml of 2% paraformaldehyde in PBS at room temperature for 10 min. Afterward, the cells were washed with 1x PBS and blocked with 3% BSA/PBS at room temperature for 1 h. The cells were stained with FITC‐conjugated anti‐CD2 antibody (Biolegend, 309206, 1:100 diluted) and APC‐conjugated anti‐CD58 antibody (Biolegend, 330918, 1:100 diluted) at 4 °C for 2 h, followed by incubation with Hoechst (Thermo, H3570, 1:10000 diluted) at room temperature for 15 min. After washed, the cells were resuspended in the mounting solution, seeded on the slides, and covered with coverslips. Images were acquired using a laser scanning confocal microscope ZEISS LSM 880 equipped with an Airyscan detector (Carl Zeiss). The co‐localization percentages of CD2 and CD58 were quantified using the Zeiss Zen 2012 (Blue Edition).

### PBMCs Isolation and CD2 Blocking Assay

Whole blood from healthy donors was collected using heparin collection tubes in accordance with the Human Subject Research Ethics, Academia Sinica, with written consent from the participants. The protocol has been approved by the Institutional Review Board of Academia Sinica (#AS‐IRB‐BM‐19058). PBMCs were isolated using Ficoll‐Paque (Cytiva, 17‐1440‐02) according to the manufacturer's instructions. T cells were then isolated using the BD IMag human T lymphocyte enrichment kit (BD, 557874). For human PBMC proliferation and CD2 blockade, PBMCs were pre‐treated with 1 µg/ml of isotype control antibody (Biolegend, 400165) or anti‐CD2 antibody (Biolegend, 309235), then treated with anti‐CD3/CD28 antibody (STEMCELL, 10971) or 1 ng ml^−1^ of recombinant wild‐type SEB. The proliferation number of the PBMCs was counted.

### Killing Assay

The killing assay was conducted using the time‐resolved immunofluorometric assay (DELFIA) (PerkinElmer, AD0116) according to the manufacturer's protocol. For the mouse T cell expansion, the splenocytes were isolated from BALB/c mice and incubated with the anti‐mouse CD3 antibody (Biolegend, 100302) and the anti‐mouse CD28 antibody (Biolegend, 102102) as the positive control, or with the 1 µg ml^−1^ SEB variants recombinant proteins for 3 days, supplemented with 100 U ml^−1^ human IL‐2 (R&D, 202‐IL‐050) and 2 ng ml^−1^ murine IL‐7 (SinoBiological, 50217‐MNAE), or 10 ng ml^−1^ Neo‐2/15. After 3 days, the cells were 1:10 diluted into a fresh culture medium supplemented with cytokines for T cell expansion. On day 5, splenocytes were washed with PBS and incubated with 5×10^3^ BATDA‐labeled mesothelin‐CT26 cells and 10 nM TTS variants recombinant proteins in the RPMI 1640 medium at 37 °C for 3 h. In the human PBMC and T cell killing assay, PBMCs and purified T cells were treated with 1 and 10 ng ml^−1^ SEB variants, respectively, in RPMI 1640 medium supplemented with 10 ng ml^−1^ Neo‐2/15. Cells were expanded and freshly supplemented with 10 ng ml^−1^ Neo‐2/15 every 3–4 days. On day 10, PBMCs and purified T cells were washed with PBS and incubated with 5 × 10^3^ BATDA‐labeled HEK‐293T or mesothelin‐ HEK‐293T cells with 10 nM SEB variants recombinant proteins in the RPMI 1640 medium at 37 °C for 3 h. The fluorescence was measured in the time‐resolved fluorometer (TECAN‐Infinite M1000 PRO).

### T Cell Proliferation Assay

For mouse T cell proliferation assay, splenocytes were isolated from BALB/c mice and incubated with the 0.1 µg ml^−1^ SEB variants recombinant proteins for 3 days. The culture medium was supplemented with 100 U/ml human IL‐2 (R&D, 202‐IL‐050) and 2 ng ml^−1^ murine IL‐7 (SinoBiological, 50217‐MNAE) or 10 ng ml^−1^ Neo‐2/15. After 3 days, the cells were 1:10 diluted into a fresh culture medium for T cell proliferation and were subcultured every 2–3 days, supplemented with the cytokines. The cells were stained with efluor780 viability dye (Thermofisher, 65–0865), CD45 (Biolegend, 103132), CD4 (Biolegend, 100516), and CD8 (Biolegend, 100725) to measure the T cell population. To evaluate the proliferation of human PBMCs, the cells were treated with 1 ng ml^−1^ SEB variant. The cells were then 1:10 diluted into a fresh culture medium for expansion and subcultured every 2–3 days, with supplementation of 10 ng ml^−1^ Neo‐2/15. To measure the T cell population, the cells were stained with efluor780 viability dye (Thermofisher, 65–0865), CD3 (Biolegend, 317306), CD4 (Biolegend, 300514), and CD8 (Biolegend, 301008).

### NK Cell Staining

Splenocytes were isolated from BALB/c mice and incubated with the SEB variants recombinant proteins for 48 h. Then the cells were stained by efluor780 viability dye (Thermofisher, 65–0865), CD45 (Biolegend, 103132), and CD335 (Biolegend, 137608). For intracellular IFN‐γ and TNF‐𝛼 staining, the cells were fixed and permeabilization using the BD Cytofix/Cytoperm Fixation/Permeabilization Kit (BD, 554714). Cells were stained with IFN‐γ (Biolegend, 505808) and TNF‐𝛼 (Biolegend, 506341) antibodies for 30 min.

### HT‐2 CCK8 Assay

5×10^4^ HT‐2 cells were seeded in 96 well plates with 200 µl of medium containing 0.83 nM of the SEB recombinant protein or Neo‐2/15, and incubated at 37 °C for 3 days. The proliferation rate of HT‐2 cells was measured using Cell Counting Kit‐8 (Dojindo).

### Mouse Tumor Experiments

BALB/c and C57BL/6 mice were provided by the National Laboratory Animal Breeding and Research Center (Taipei, Taiwan). All animal studies were conducted in specific pathogen‐free conditions and in accordance with guidelines approved by the Animal Care and Usage Committee of Academia Sinica and housed at a temperature of 19–23 °C under a 12‐h light‐dark cycle and a humidity of 50‐ 60%. A maximum of five mice were housed in a single individually ventilated cage with soft wood for nesting. To establish the syngeneic mouse tumor model, 1×10^6^ cancer cells were suspended in 100 µl RPMI 1640 or DMEM medium and were subcutaneously injected at the right flank of each mouse for 5–6 days. when the tumor volume reached ≈50–100 mm^3^, 5 µg of proteins were dissolved in 80 µl PBS and administered via intravenous injection using insulin syringes, followed by two additional injections of 1 µg protein each on day 6 and day 12.

### Tumor‐Infiltrating Leukocytes Analysis

The tumors were dissociated by 0.3 mg ml^−1^ collagenase IV (Sigma–Aldrich, C5138) and 0.06 mg ml^−1^ DNaseI (Cyrusbioscience, 101‐9003‐98‐9) in the RPMI 1640 medium supplemented with 10% FBS (v/v). Cells were then incubated with the RBC lysis buffer (Biolegend, 420301), followed by filtering through a 40 µm cell strainer. The cells were blocked by the anti‐mouse CD16/32 antibody (Biolegend, 101301) in the FACS buffer before immunostaining. The cells were stained by efluor780 viability dye (Thermofisher, 65–0865), CD45 (Biolegend, 103132), CD4 (Biolegend, 100516), CD8 (Biolegend, 100725), MHC‐II (Biolegend, 107606), CD11b (Biolegend, 101206), Ly6G (Biolegend, 129614), F4/80 (Biolegend, 123130), CD335 (BD Biosciences, 562850). The CD11b and Ly6G were used as the neutrophil marker. The MHC‐II and F4/80 were used as the macrophage marker. The stained cells were analyzed by flow cytometry (Attune NxT) cytometer, and the data were processed by FlowJo V10 software.

### Pharmacokinetics Study and Tumor Accumulation Analysis

Recombinant proteins were labeled with Alexa Fluor 647 NHS Ester (Thermo, A20006) at a ratio of 2 µg dye per 1 µg protein and incubated in d_2_H_2_O for 2 h at room temperature. Subsequently, the unconjugated dye was removed using protein concentrators (Thermo, 88513). Following this, 5 µg of fluorescence‐labeled recombinant proteins were intravenously injected into BALB/c mice. Serum samples were collected starting from 10 min post‐injection and assessed at various time points to measure the fluorescence levels. For the tumor accumulation measurement, 5 µg of fluorescence‐labeled recombinant proteins were intravenously injected into BALB/c mice bearing meso‐CT26 tumors ranging from 50–100 mm^3^ in size. After 24 h, tumors were homogenized twice for 20 s. Subsequently, the homogenates were centrifuged at 2000 g for 5 min, and the supernatant was collected to measure fluorescence levels.

### Histology Analysis

All mice were sacrificed on day 2 after the second protein injection. To assess the in vivo safety of STYMIE, mice were perfused with PBS and 4% formalin, embedded in paraffin and stained by hematoxylin and eosin (H&E). The slides were scanned using the PANNORAMIC 250 slide scanner (3DHistech) and analyzed using CaseViewer 2.4 (3DHistech).

### Surface Plasmon Resonance Spectroscopy

SEB proteins were diluted to 10 in 10 mM Na acetate, pH 5.0, and immobilized on a CM5 sensorchip (Cytiva) using an amine coupling kit and amine‐thiol coupling kit (BIAcore), respectively. Analytes human CD28‐Fc (SinoBiological, 11524‐H02H) were injected at 30 µL min^−1^ in PBS, pH 7.4, 3.4 mM EDTA, and 0.005% surfactant P20. Kinetic analyses were performed at 25 °C in a BIAcore T200 instrument, deducting the control flow cell signal from the binding signal. The maximum response unit (Rmax) was controlled to be below 30 to reduce mass transport limitations according to the manufacturer protocol. A double serial dilution of human CD28‐Fc was performed in PBSP buffer from 5.2 µm to 0.325 µm and flowed passed (30 µl min^−1^) the immobilized chip with an association and dissociation time of 150 and 600s respectively. The binding affinity (KD) and dissociation constants (Kd) were calculated using Biacore T200 evaluation software and the resulting binding curves were regenerated using GraphPad Prism 8.0.

### Statistical Analysis

Prism 8 (GraphPad Software) was used for statistical analysis. p‐value significance was calculated using the One‐way ANOVA, Two‐way ANOVA or Student's *t*‐test. A p‐value under 0.05 was regarded as statistically significant. The error bars in the figures represent mean ± SD.

## Conflict of Interest

The authors declare no conflict of interest.

## Author Contributions

Y.A.Y., C.M.H., and K.Y.M. conceived and designed the experiments. Y.A.Y., W.J.L., W.C.L., Y.C.P., and S.W.H. performed the experiments. Y.A.Y., C.Y.M., C.M.H., and K.Y.M. discussed the experimental results and wrote the manuscript. All authors provided clarification, guidance, and revision of the manuscript.

## Supporting information

Supporting Information

## Data Availability

The data that support the findings of this study are available from the corresponding author upon reasonable request.
