# Supplementary material for: High‐Affinity Superantigen‐Based Trifunctional Immune Cell Engager Synergizes NK and T Cell Activation for Tumor Suppression
Source: Adv Sci (Weinh). 2024 Jun 27;11(33):2310204. doi: 10.1002/advs.202310204 (PMC11434130; doi:10.1002/advs.202310204)
Supplement: Supplementary file 1 — Supporting Information [file ADVS-11-2310204-s001.docx]

**Supplementary Information**

**High-Affinity Superantigen-Based Trifunctional Immune Cell Engager Synergizes NK and T Cell Activation for Tumor Suppression**

**Yao-An Yu^1,2^, Wan-Ju Lien^1^, Wen-Ching Lin^1^, Yi-Chung Pan^1^, Sin-Wei Huang^1^, Chung-Yuan Mou^4^, Che-Ming Jack Hu^1,2,3*^, and Kurt Yun Mou^♱^**

1 Institute of Biomedical Sciences, Academia Sinica, Taipei, 11529, Taiwan.
2 Doctoral Degree Program of Translational Medicine, National Yang Ming Chiao Tung University and Academia Sinica, Taipei 112, Taiwan.

^3^ Biomedical Translation Research Center, Academia Sinica, Taipei, 11529, Taiwan.

^4^ Department of Chemistry, National Taiwan University, Taipei 10617, Taiwan.

^♱^ The author passed away on August 28^th^, 2023.

***To whom correspondence should be addressed:**

Che-Ming Jack Hu, Ph.D.

Institute of Biomedical Sciences, Academia Sinica, Taipei, 11529, Taiwan.

Phone: +1-886-2-26523089

Fax: +1-886-2-27887641

Email: chu@ibms.sinica.edu.tw

**List of supplementary figures:**

Figure S1 | Structural analysis of the wild-type SEB and the mutated SEB from the S4 library.

Figure S2 | The D55G mutation on SEB impaired NFAT signaling activation.

Figure S3 | The expression levels of the immunoreceptors on the Jurkat-hTCRvβ3 CRISPR knockout cells.

Figure S4 | The structural prediction of the protein-protein interaction of SEB and human CD2 using AlphaFold2-Multimer.

Figure S5 | The structural prediction of the protein-protein interaction of SEB and human CD58 using AlphaFold2-Multimer.

Figure S6 | The structural prediction of the protein-protein interaction of SEB and mouse CD2 using AlphaFold2-Multimer.

Figure S7 | The structural prediction of the protein-protein interaction of SEB and mouse CD48 using AlphaFold2-Multimer.

Figure S8 | The TCR is required for SEB-induced NFAT signaling activation.

Figure S9 | Functional characterization of the TF proteins.

Figure S10 | The absorbance (280 nm) profile of Fc proteins eluted from the FPLC.

Figure S11 | Tumor growth curve of individual mice within each treatment group.

Figure S12 | Surface plasmon resonance spectroscopy examining superantigen binding affinity with CD28.

Figure S13 | TILs analysis of excised tumors from each treatment group on day 18 after the first protein injection.

Figure S14 | Tumor weight of the mesothelin-CT26 tumor-bearing mice in different treatment groups.

Figure S15 | Tumor growth curve of individual mice within each treatment group.

Figure S16 | Tumor weight of the mesothelin-4T1 and mesothelin-LLC tumor-bearing mice in different treatment groups.

Table S1 | Comprehensive blood analysis following intravenous STYMIE administration.


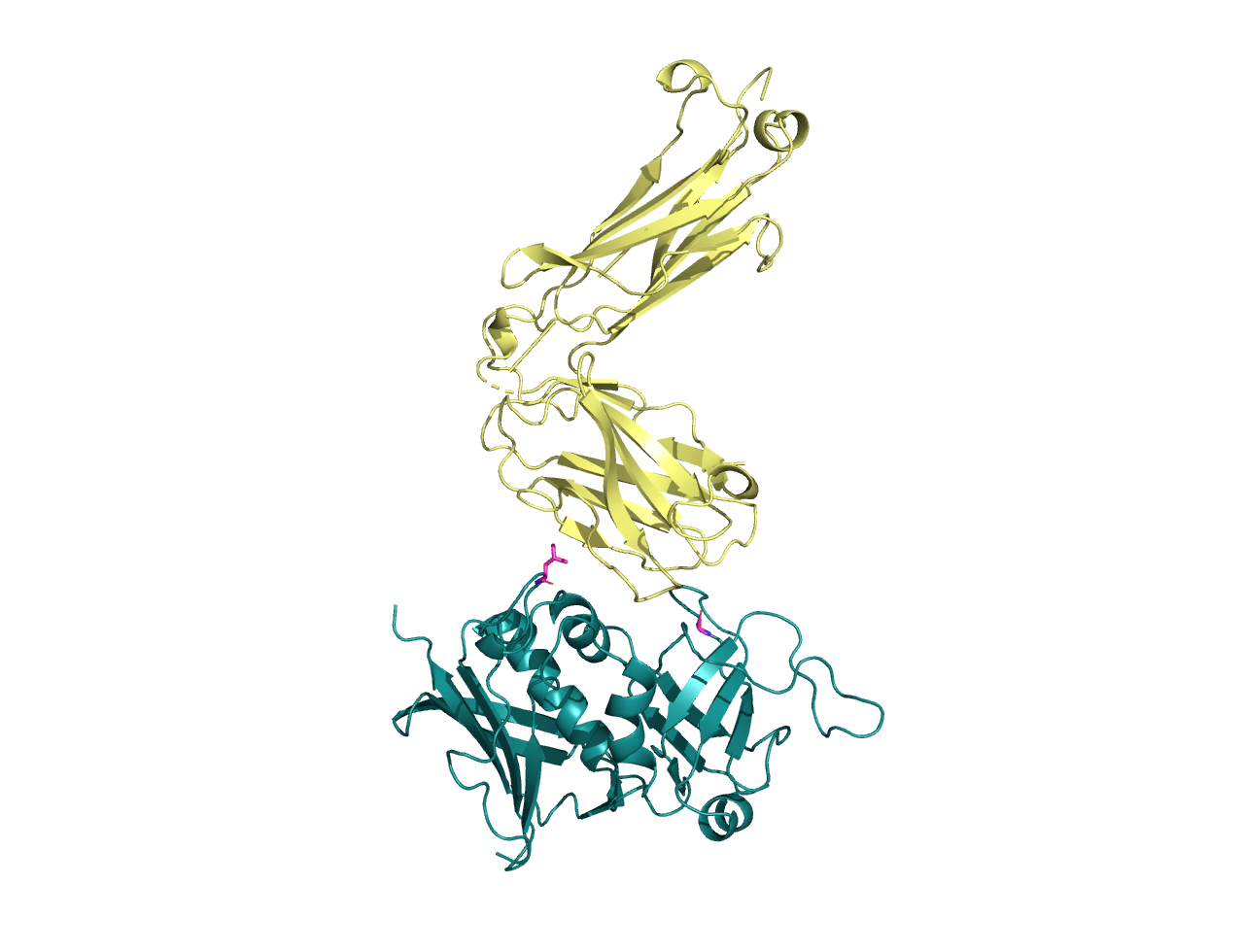


**TCR**

**S4 SEB**

**F177L**

**D55G**

**a**

**b**


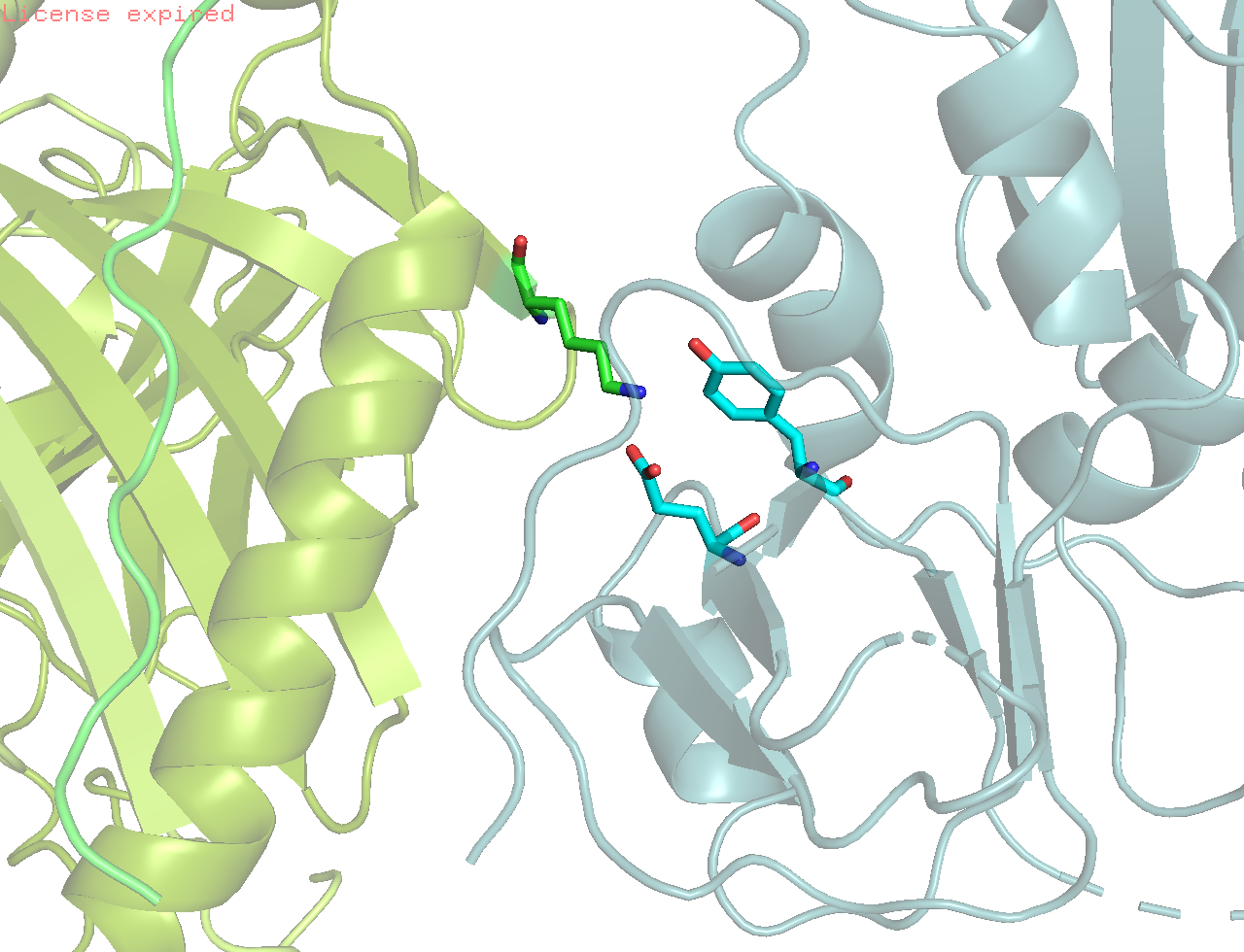


**HLA-DR**

**K39**

**E67**

**Y115**

**WT SEB**


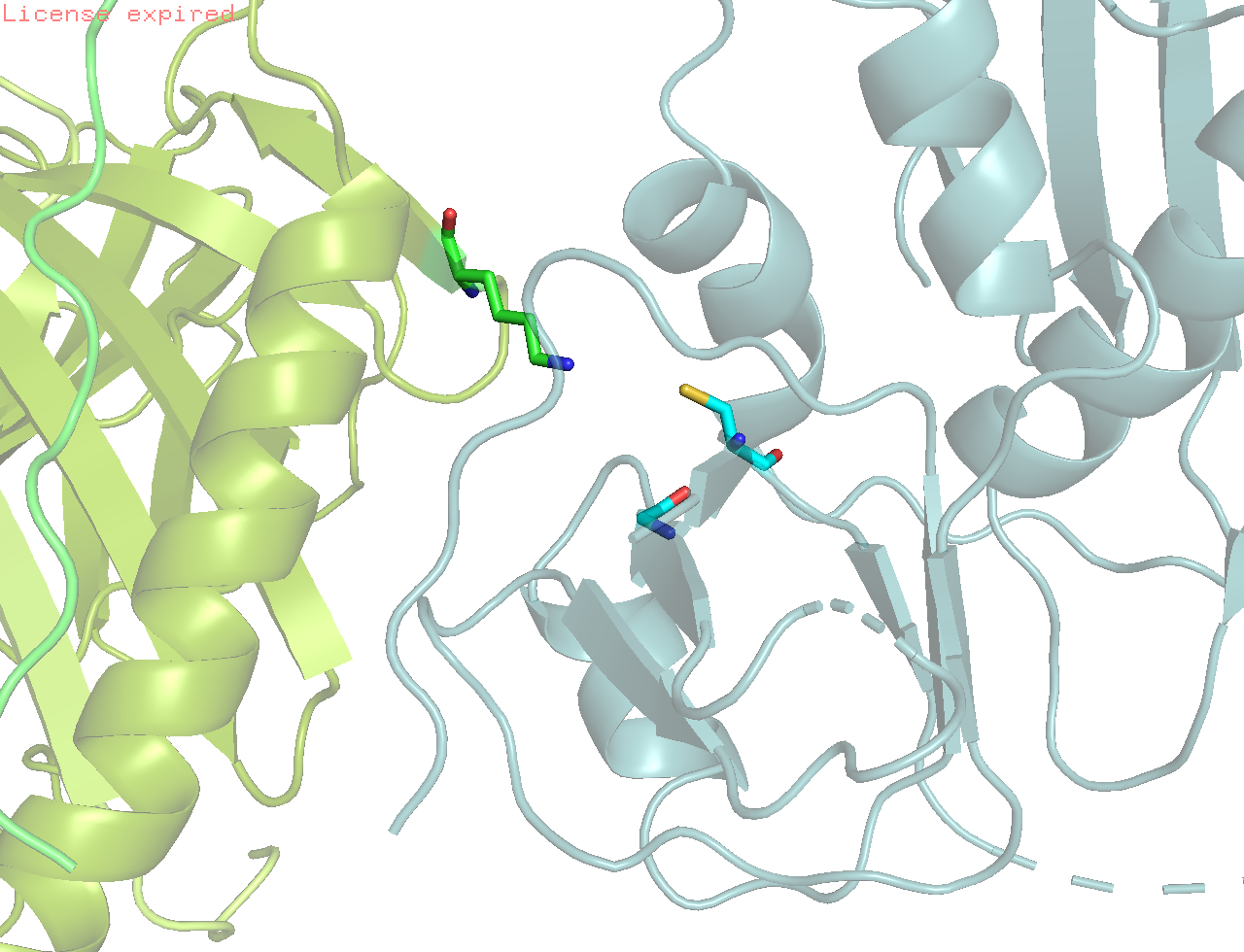


**S4 SEB**

**HLA-DR**

**K39**

**E67G**

**Y115C**


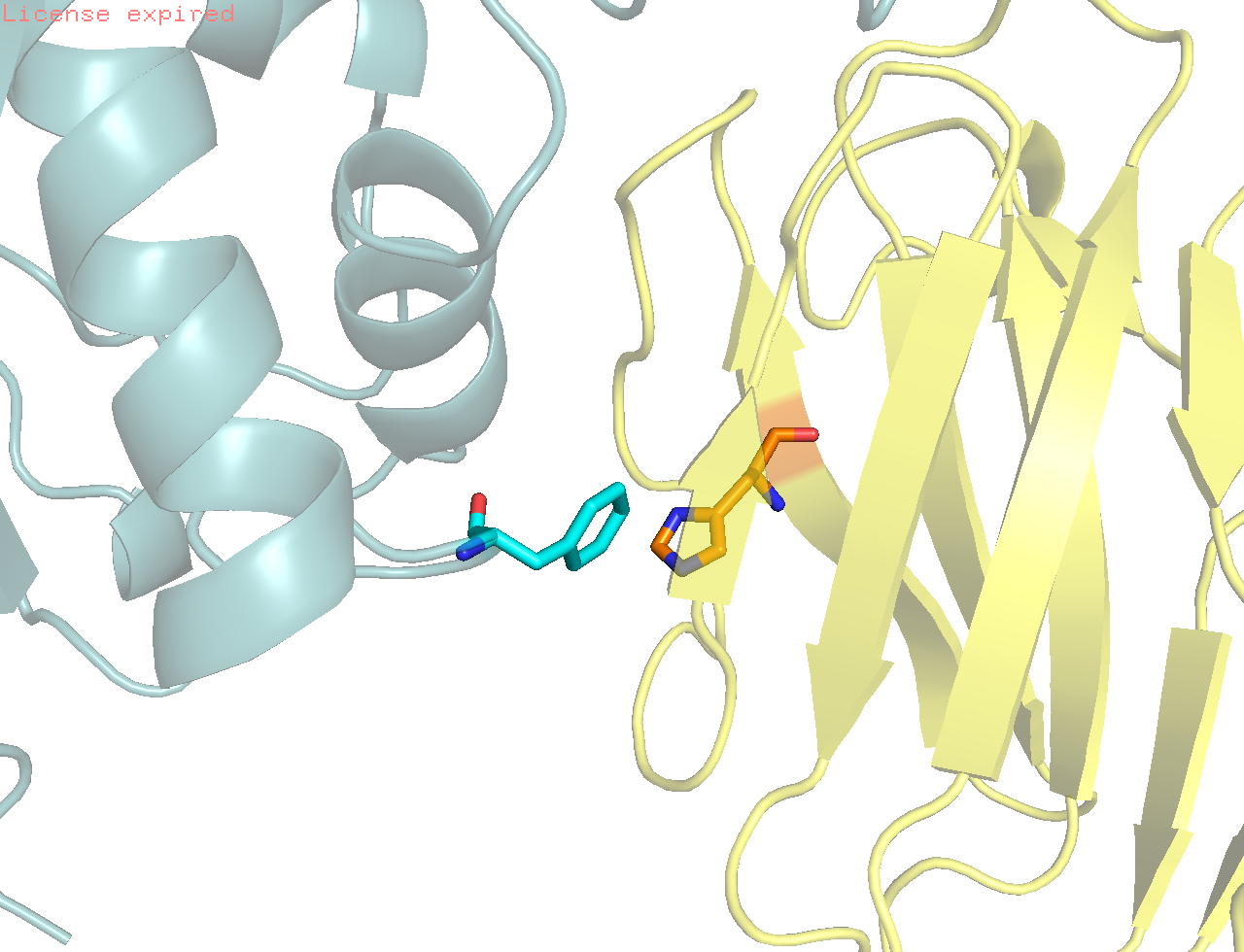


**WT SEB**

**TCR**

**F177**

**H47**


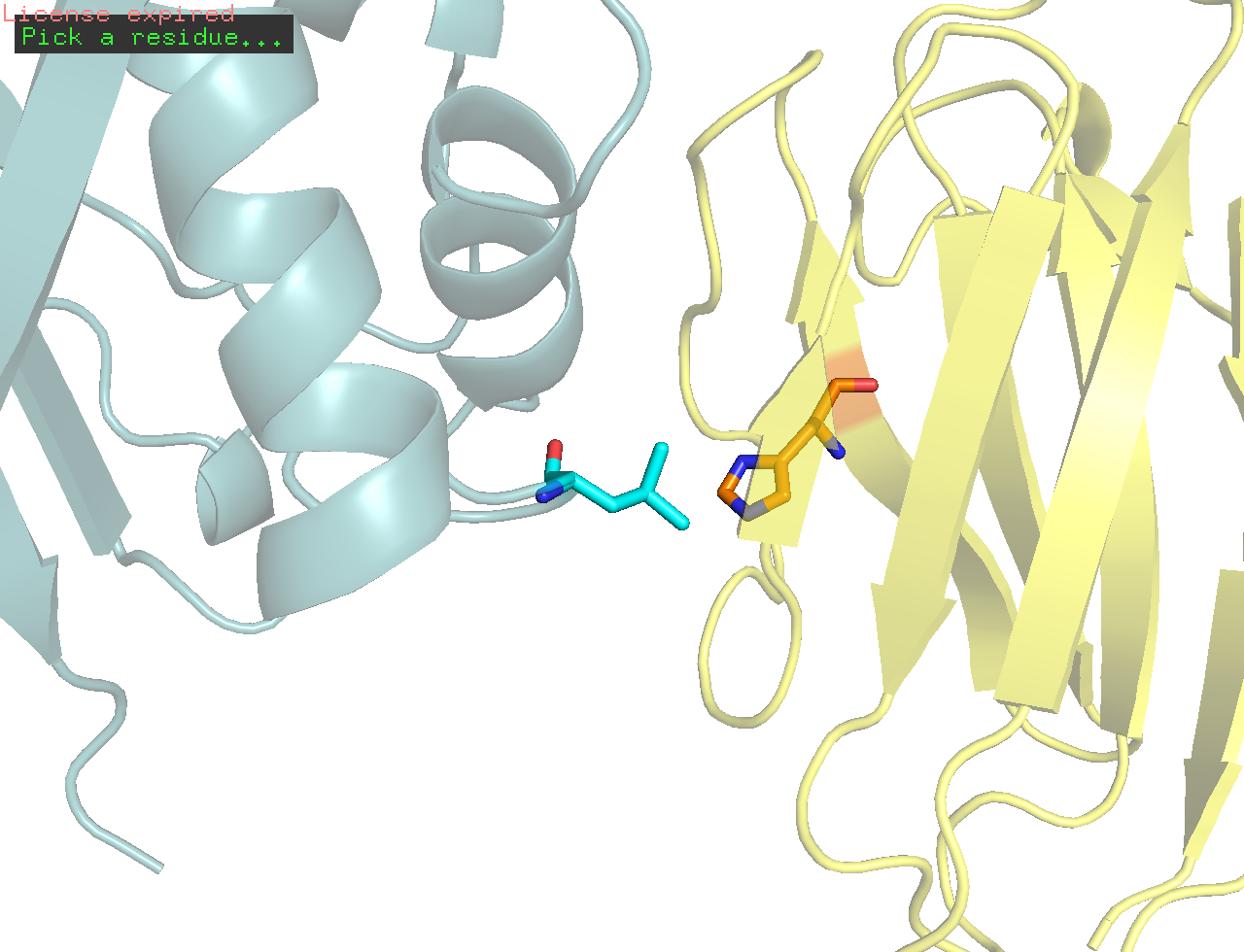


**S4 SEB**

**TCR**

**F177L**

**H47**

**c**

**d**


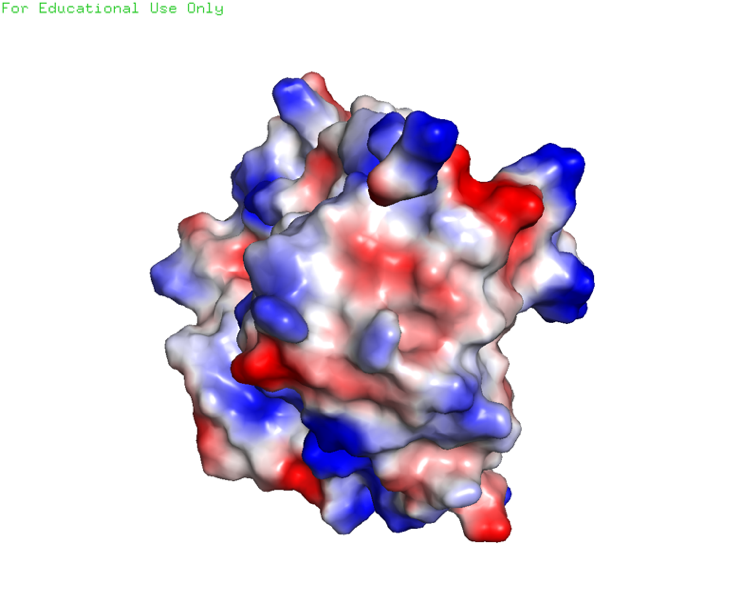


**S4 SEB**

**D101K**

**K71R**

**K78R**

**Figure S1 | Structural analysis of the wild-type SEB and the mutated SEB from the S4 library.** The protein-protein interaction interfaces of (a) SEB-HLA-DR and (b, c) SEB-TCR. (d) The surface electrostatic potential of SEB from S4 library (S4 SEB) was calculated by PyMOL. The positive charge was shown in blue and the negative charge was shown in red.

**
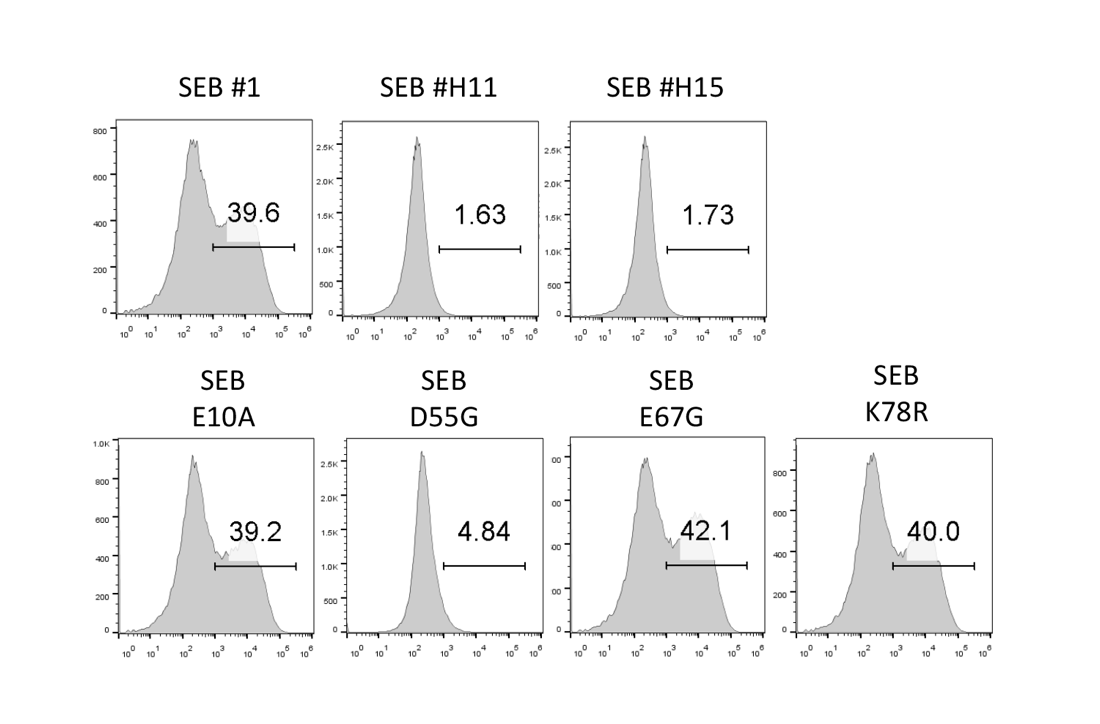
**

**Figure S2 | The D55G mutation on SEB impaired NFAT signaling activation.** The Jurkat-hTCRvβ3 NFAT-GFP reporter cells were treated with 1 μg/ml of recombinant wild-type SEB or SEB variants. After 24 hours, the percentage of activated NFAT-GFP signaling was determined using flow cytometry.


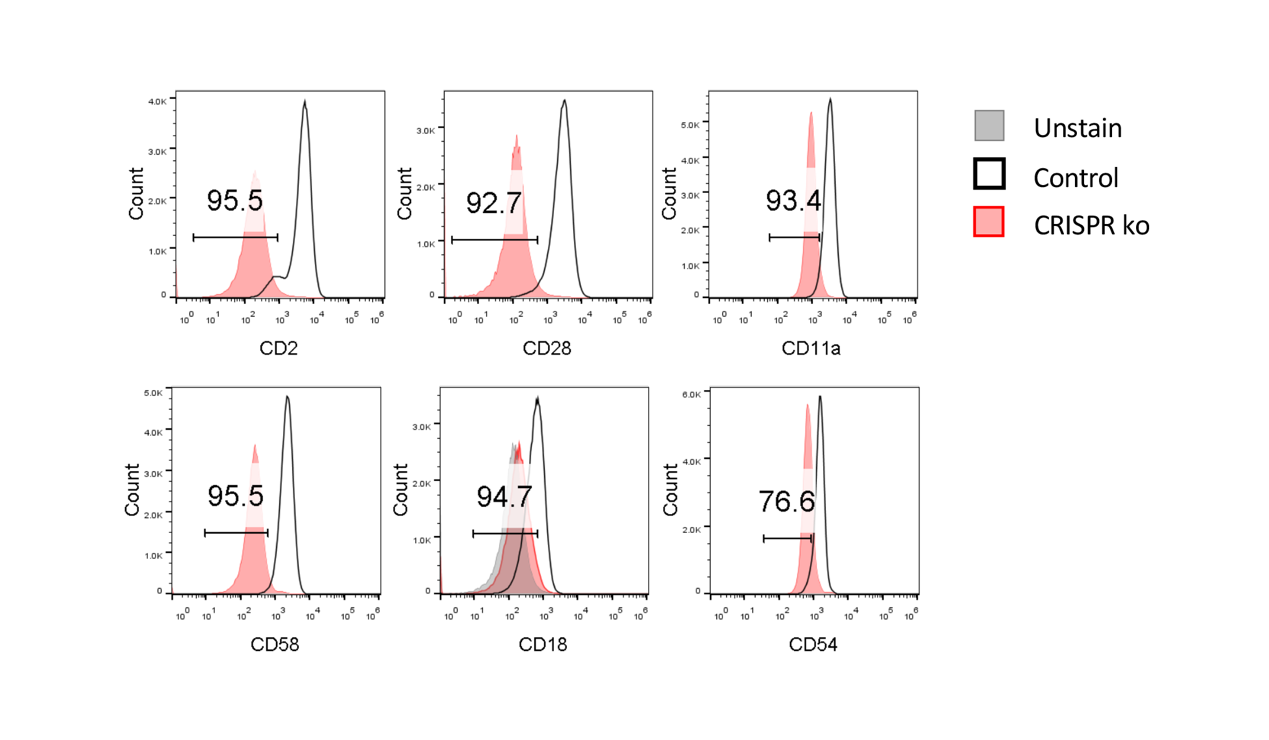


**Figure S3 | The expression levels of the immunoreceptors on the Jurkat-hTCRvβ3 CRISPR knockout cells.** The expression levels of the immunoreceptors on the Jurkat-hTCRvβ3 NFAT-GFP reporter cells and the immunoreceptors CRISPR knockout efficiency were measured using flow cytometry.

**Figure S4 | The structural prediction of the protein-protein interaction of SEB and human CD2 using AlphaFold2-Multimer.** (a-d) The SEB-hCD2 protein complex is predicted using the AlphaFold2-Multimer. The predicted 25 models were superimposed and divided into several clusters based on their structural similarity.

**Figure S5 | The structural prediction of the protein-protein interaction of SEB and human CD58 using AlphaFold2-Multimer.** (a-d) The SEB-hCD58 protein complex is predicted using the AlphaFold2-Multimer. The predicted 25 models were superimposed and divided into several clusters based on their structural similarity.

**Figure S6 | The structural prediction of the protein-protein interaction of SEB and mouse CD2 using AlphaFold2-Multimer.** (a-d) The SEB-mCD2 protein complex is predicted using the AlphaFold2-Multimer. The predicted 25 models were superimposed and divided into several clusters based on their structural similarity.

**Figure S7 | The structural prediction of the protein-protein interaction of SEB and mouse CD48 using AlphaFold2-Multimer.** (a-d) The SEB-mCD48 protein complex is predicted using the AlphaFold2-Multimer. The predicted 25 models were superimposed and divided into several clusters based on their structural similarity.

**Figure S8 | The TCR is required for SEB-induced NFAT signaling activation.** The Jurkat-hTCRvβ3 NFAT-GFP reporter cells and Jurkat TCR𝛼/β-/- NFAT-GFP reporter cells were treated with 1 μg/ml of recombinant wild-type SEB. After 24 hours, the percentage of activated NFAT-GFP signaling was determined using flow cytometry.

**
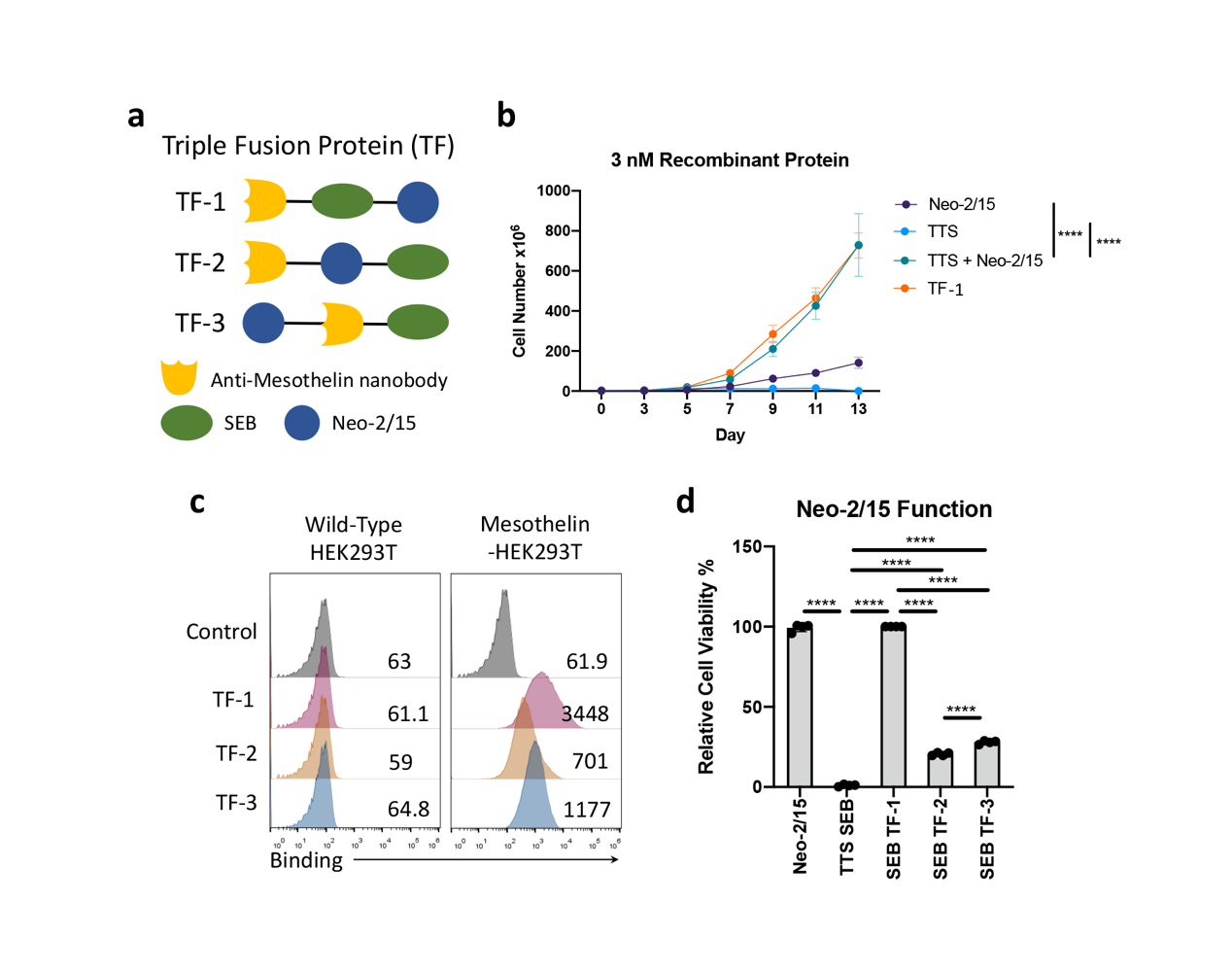
 Figure S9| Functional characterization of the TF proteins.** (a) Schematic representation of the TF protein design. (b) Mouse splenocytes were stimulated with 3 nM of recombinant TTS with or without 3 nM Neo-2/15 supplementation, or with 3 nM of TF-1 protein. The cells were subcultured every 3 days and supplemented with fresh recombinant proteins. Cell numbers were counted for every 2 days. (n=3) (c) 10 nM of recombinant TF proteins were co-incubated with both wild-type HEK293T and mesothelin-HEK293T cells. The ability of the anti-mesothelin nanobody to bind to mesothelin was confirmed by flow cytometry. (d) HT-2 cells were treated with 0.83 nM of Neo-2/15, recombinant TTS, or TF proteins in 96-well plates. The viability of the HT-2 cells was measured after 3 days using CCK-8. (n=3) The error bars represent mean ± SD. Statistical analyses were performed by (b) Two-way ANOVA with Tukey correction and (d) One-way ANOVA with Tukey correction. (**** *p* <0.0001).

**Supplementary Fig. 10 | The absorbance (280 nm) profile of Fc proteins eluted from the FPLC.** The protein purity was examined by size-exclusion chromatography using a Superdex 200 increase column (GE Healthcare) in PBS.

**Figure S11 | Tumor growth curve of individual mice within each treatment group.** The tumor growth curve of mesothelin-CT26 tumor-bearing mice in different treatment groups. (n=5 to 12)

**Figure S12 | Surface plasmon resonance spectroscopy examining superantigen binding affinity with human CD28.** Comparison between SEB #1 and SEB #30 shows SEB #30’s enhanced human CD28 affinity as a result of directed evolution.

**Figure S13 | TILs analysis of excised tumors from each treatment group on day 18 after the first protein injection.** The level of neutrophils (CD45^+^CD11b^+^Ly6G^+^), macrophages (CD45^+^F4/80^+^), and B cells (CD45^+^CD19^+^) was determined via flow cytometry. (n=6 to 11)

**Figure S14 | Tumor weight of the mesothelin-CT26 tumor-bearing mice in different treatment groups. (n=5 to 14)**

**Figure S15 | Tumor growth curve of individual mice within each treatment group.** (a) The tumor growth curve of mesothelin-4T1 (n=6 to 8) and (b) mesothelin-LLC tumor-bearing mice in different treatment groups. (n=5 to 7)

**Figure S16 | Tumor weight of the mesothelin-4T1 (n=5 to 8) and mesothelin-LLC tumor-bearing mice (n=5 to 7) in different treatment groups.**

**
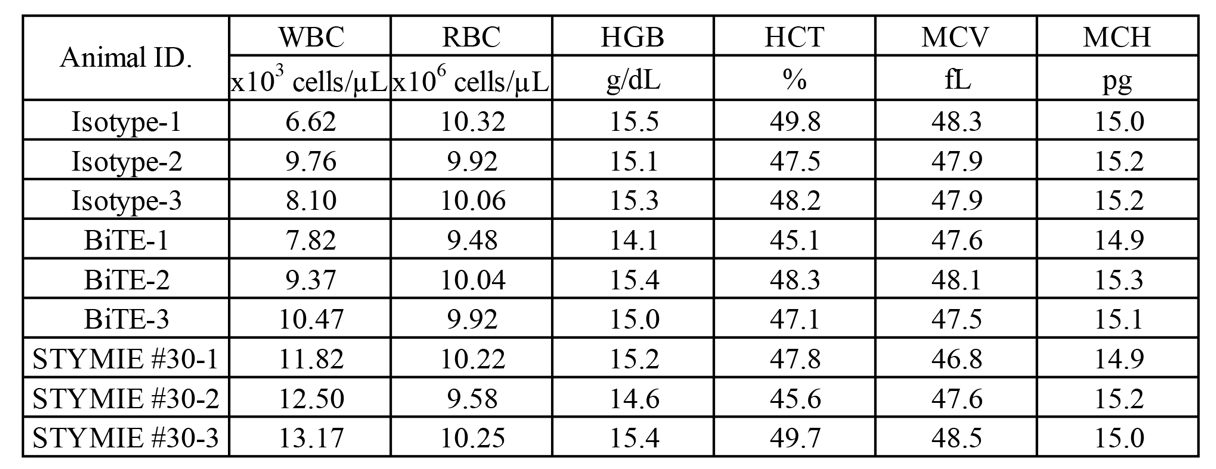
**

**
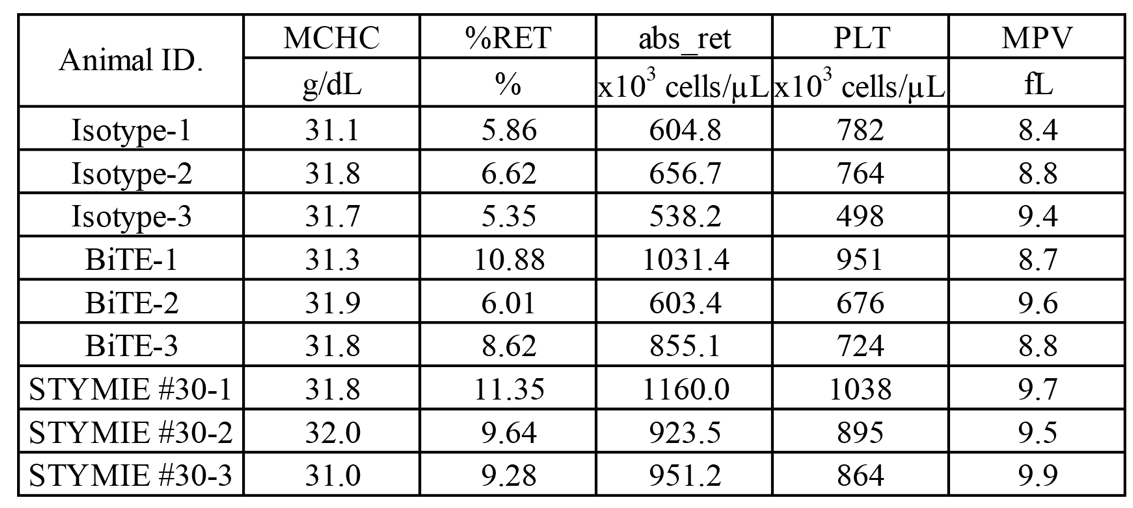
**

**
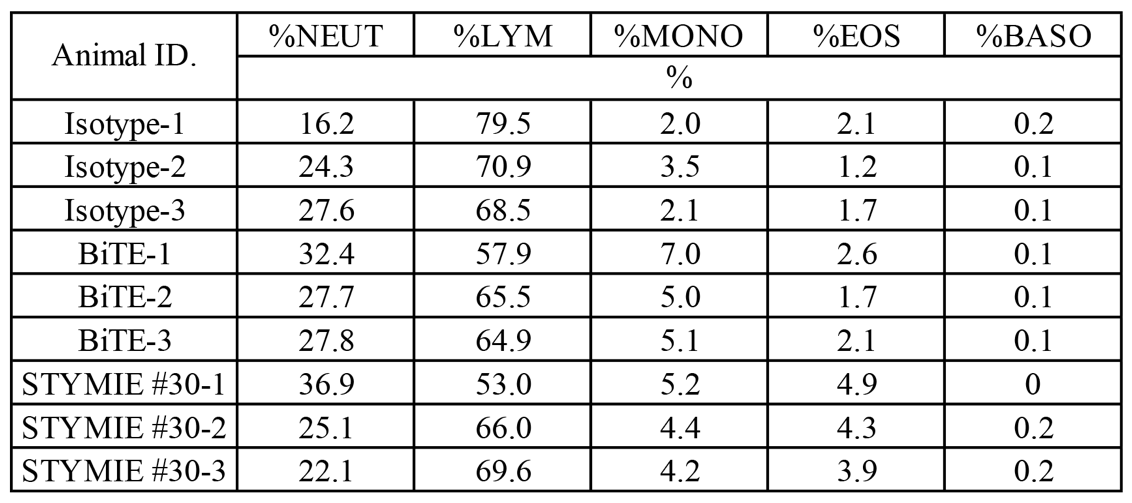
**

**
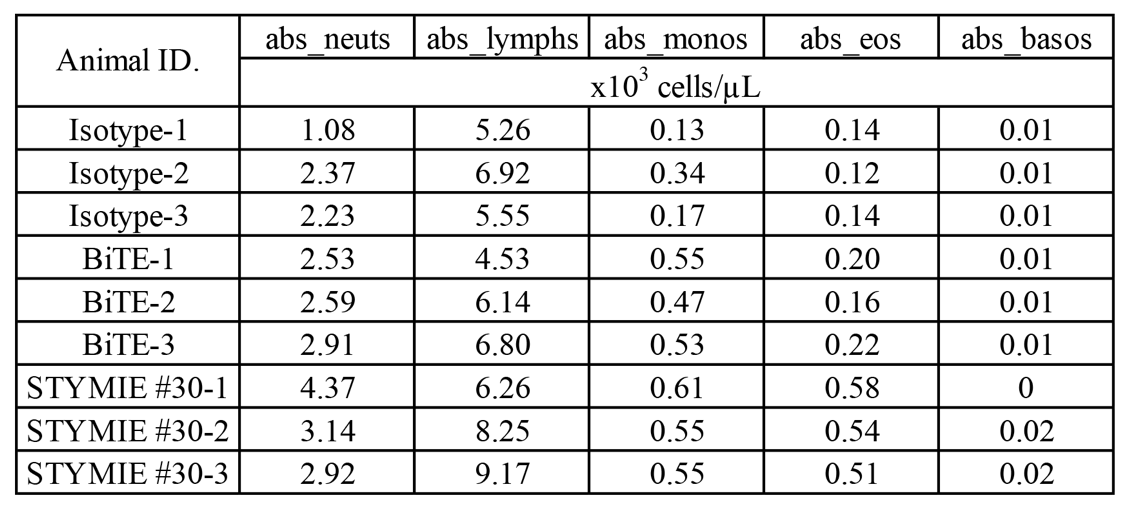
**

**Table S1 | Comprehensive blood analysis following intravenous STYMIE administration.** Hematology analyses were conducted using whole blood samples from mice on the second day following the administration of the second dose of Isotype control antibody, BiTE, and STYMIE #30. (n=3) WBC (white blood cells), RBC (red blood cells), HGB (hemoglobin), HCT (Hematocrit), MCV (Mean corpuscular volume), MCH (Mean corpuscular hemoglobin), MCHC (Mean corpuscular hemoglobin concentration), PLT (Platelet), MPV (Mean Platelet Volume), % (percent), abs (absolute counts), NEUT (neutrophils), LYM (lymphocytes), MONO (monocytes), EOS (eosinophils), BASO (basophils), RET (reticulocyte).
